# Activation of aryl hydrocarbon receptor (AhR) in Alzheimer’s disease: role of tryptophan metabolites generated by gut host-microbiota

**DOI:** 10.1007/s00109-023-02289-5

**Published:** 2023-02-09

**Authors:** Antero Salminen

**Affiliations:** Department of Neurology, Institute of Clinical Medicine, University of Eastern Finland, P.O. Box 1627, Kuopio, 70211 Finland

**Keywords:** Aging, Hypoperfusion, Immunosuppression, Microbiome, Microflora, Uremic toxin

## Abstract

**Abstract:**

Gut microbiota in interaction with intestinal host tissues influences many brain functions and microbial dysbiosis has been linked with brain disorders, such as neuropsychiatric conditions and Alzheimer’s disease (AD). l-tryptophan metabolites and short-chained fatty acids (SCFA) are major messengers in the microbiota-brain axis. Aryl hydrocarbon receptors (AhR) are main targets of tryptophan metabolites in brain microvessels which possess an enriched expression of AhR protein. The Ah receptor is an evolutionarily conserved, ligand-activated transcription factor which is not only a sensor of xenobiotic toxins but also a pleiotropic regulator of both developmental processes and age-related tissue degeneration. Major microbiota-produced tryptophan metabolites involve indole derivatives, e.g., indole 3-pyruvic acid, indole 3-acetaldehyde, and indoxyl sulfate, whereas indoleamine and tryptophan 2,3-dioxygenases (IDO/TDO) of intestine host cells activate the kynurenine (KYN) pathway generating KYN metabolites, many of which are activators of AhR signaling. Chronic kidney disease (CKD) increases the serum level of indoxyl sulfate which promotes AD pathogenesis, e.g., it disrupts integrity of blood–brain barrier (BBB) and impairs cognitive functions. Activation of AhR signaling disturbs vascular homeostasis in brain; (i) it controls blood flow via the renin-angiotensin system, (ii) it inactivates endothelial nitric oxide synthase (eNOS), thus impairing NO production and vasodilatation, and (iii) it induces oxidative stress, stimulates inflammation, promotes cellular senescence, and enhances calcification of vascular walls. All these alterations are evident in cerebral amyloid angiopathy (CAA) in AD pathology. Moreover, AhR signaling can disturb circadian regulation and probably affect glymphatic flow. It seems plausible that dysbiosis of gut microbiota impairs the integrity of BBB via the activation of AhR signaling and thus aggravates AD pathology.

**Key messages:**

- Dysbiosis of gut microbiota is associated with dementia and Alzheimer’s disease.
- Tryptophan metabolites are major messengers from the gut host-microbiota to brain.
- Tryptophan metabolites activate aryl hydrocarbon receptor (AhR) signaling in brain.
- The expression of AhR protein is enriched in brain microvessels and blood-brain barrier.
- Tryptophan metabolites disturb brain vascular integrity via AhR signaling.
- Dysbiosis of gut microbiota promotes inflammation and AD pathology via AhR signaling.

## Introduction

The human microbiome has been commonly referred to as our second genome since it influences many functions of the host organism [1]. Microbiota involves a diversity of microorganisms, such as bacteria, e.g., *Actinobacteria*, *Bacteroidetes*, *Firmicutes*, *Fusobacteria*, and *Proteobacteria*, as well as fungi, archaea, and viruses, which colonize several loci of the body, e.g., gut, skin, mouth, and nares [1]. The gut houses the largest population of microorganisms which secrete a number of messenger molecules, such as l-tryptophan metabolites, short-chain fatty acids (SCFA), secondary bile acids, and peptidoglycan fragments [2]. Currently, there is compelling evidence that dysbiosis of the gut microbiota is involved in the pathogenesis of neurodegenerative diseases, such as Alzheimer’s disease (AD) [3–5].

Many tryptophan metabolites and SCFA produced by gut microbiota in a close interaction with intestine host cells activate aryl hydrocarbon receptor (AhR) [6–8]. The ligand-activated AhR transcription factor is not only an evolutionarily conserved sensor of xenobiotic toxins but also a pleiotropic regulator of both developmental differentiation and many age-related degenerative processes [9]. There are studies indicating that the expression of AhR protein was robustly increased in the post-mortem hippocampus of AD patients as compared to the level of cognitively healthy elderly people [10]. It is known that the expression of AhR protein is particularly abundant in vascular tissues and it has a crucial role in the vascular diseases [11]. Interestingly, the integrity of the blood–brain barrier (BBB) is impaired in AD patients and it seems that the disturbances in the BBB might enhance AD pathogenesis [12, 13]. First, I will review the evidences indicating that the gut host-microbiota/brain axis driven by tryptophan metabolites has an important role in AD pathogenesis. Next, I will examine in detail the role of AhR signaling activated by tryptophan metabolites in cerebral vascular disturbances and the loss of BBB integrity.

## Activation of AhR signaling

The AhR protein is an evolutionarily conserved member of an ancient basic helix-loop-helix/PER-ARNT-SIM (bHLH/PAS) family. The Ah receptor is a ligand-regulated transcription factor which evolved over 600 million years ago [14]. The Ah receptor is a major environmental sensor for a number of xenobiotic toxins, such as 2,3,7,8-tetrachlorodibenzo-p-dioxin (TCDD) and polycyclic aromatic hydrocarbon (PAH) compounds [15]. Moreover, there are several phytochemicals that can act as either agonists or antagonists for Ah receptor [16]. For example, chrysin, baicalein, and genistein are potent agonists for AhR signaling, whereas resveratrol, luteolin, and curcumin are effective antagonists. The Ah receptor also recognizes several naturally occurring endogenous ligands including many metabolites of l-tryptophan as well as some prostaglandins and modified low-density lipoproteins [15, 17]. The crucial tryptophan metabolites, especially those generated by microbiota, will be addressed below in a detailed manner. In the cytoplasm, the AhR protein exists in a complex with the chaperone proteins, i.e., HSP90, p23, and XAP2 [18]. After the binding of an agonist, Ah receptor is translocated into the nucleus where it forms a heterodimer with the AhR nuclear translocator (ARNT) protein, also a member of bHLH/PAS family. The AhR/ARNT complex binds to the dioxin/xenobiotic response element (DRE/XRE) in the promoter sequences of AhR-responsive genes [18]. There is also an endogenous inhibitor for AhR signaling, i.e., the AhR repressor (AhRR) protein, which does not contain the transactivation domain [19]. However, the expression level of AhRR protein is low in human brain (Human Protein Atlas). In addition, AhR protein can arrange DNA-binding complexes with RelB and E2F1 transcription factors [20, 21] but these complexes do not bind to the DRE sites and do not transactivate AhR-responsive genes. The activated Ah receptor can also stimulate a non-genomic signaling by activating Src kinase which subsequently can trigger other signaling pathways, e.g., focal adhesion kinase (FAK) [22, 23].

AhR signaling has a significant role in the protection of organisms against environmental toxins, i.e., Ah receptor controls the detoxification of xenobiotic compounds [15]. In particular, AhR signaling has important functions in barrier organs, such as the gut, skin, and lungs [24]. For instance, Ah receptor regulates the immune homeostasis in the gut, and thus, it is important in defence against infections [24, 25]. Moreover, it maintains tolerance against harmless antigens, i.e., both dietary and microbiota-generated antigens. Interestingly, AhR signaling displays evidence of a process called antagonistic pleiotropy which means that Ah receptor has an important role in embryogenesis but later in life it promotes age-related degenerative processes [9]. For instance, Ah receptor regulates the differentiation of embryonal stem cells [26]. AhR signaling also controls the proliferation and differentiation of hematopoietic stem cells and progenitor cells [27], and in this way, it maintains the homeostasis of the immune system, e.g., in chronic inflammatory conditions. AhR signaling is also able to enhance neurogenesis. Wei et al. [28] demonstrated that tryptophan metabolites originating from gut microbiota could stimulate neurogenesis in adult mouse hippocampus in an AhR-dependent manner. On the other hand, there is substantial evidence that AhR signaling promotes many age-related degenerative processes [9]. For instance, it is known that AhR signaling inhibits autophagy [29, 30], enhances cellular senescence [30], evokes disturbances in the extracellular matrix [31], and can provoke vascular dystrophy [32]. These are all common hallmarks of the aging process. Recently, Ojo and Tischkau [33] reviewed the findings indicating the clear association of AhR signaling with many of the hallmarks of aging in the brain and also in age-related brain diseases.

## Microbiota gut-brain axis in AD pathogenesis

There is substantial evidence that the microbiota of the gut has a crucial role in many functions of brain physiology and pathology [34]. Furthermore, there are bidirectional interactions between the gut and the brain since the brain can control the metabolism of microbiota through the branches of the vagus nerve and the hypothalamus-pituitary-adrenocortical (HPA) axis [35, 36]. Moreover, it is recognized that the gut microbiota regulates the intestinal immunity, e.g., colonic inflammation, thus affecting the function of the gut barrier and host immunity [37]. Given that tryptophan metabolites and SCFAs control the functions of Ah receptors (see below), it seems that AhR signaling has an important role in the communication between the microbiome and brain metabolism and pathology [7, 8].

There is robust evidence that dysbiosis in the gut microbiota is associated with many common diseases, e.g., atherosclerosis, diabetes, metabolic syndrome, inflammatory bowel disease, and central nervous system disorders [38]. In particular, gut microbial metabolites affect the homeostasis of the brain inducing neuropsychiatric disorders and promoting neurodegenerative diseases, such as AD, multiple sclerosis, and Parkinson’s disease [39]. Recently, several review articles have examined in detail the experiments indicating that the microbiota-induced neuroinflammation and neurodegeneration promote the pathogenesis of AD [3–5]. Wu et al. [40] demonstrated that there existed significant differences in gut microbial metabolites between the healthy controls and patients with either mild cognitive impairment (MCI) or AD. In particular, the tryptophan-derived metabolites of serotonin pathway were at significantly lower level in the fecal samples of MCI and AD patients as compared to those of healthy controls. For instance, there were differences in the amounts of indole derivatives, e.g., the level of indole-3-pyruvic acid (IPA), a potent activator of AhR signaling [41], was robustly upregulated, whereas those of 5-hydroxyindole and indole 2-carboxylic acid were downregulated. Wu et al. [40] also revealed that an increased cognitive impairment positively correlated with reduced metabolites of serotonin pathway, whereas there was a negative correlation between the degree of cognitive impairment and the level of IPA. Several studies have revealed that the concentrations of tryptophan and serotonin are significantly reduced in the serum of AD patients [42, 43] which might indicate that there is increased tryptophan catabolism in the gut. Accordingly, Teruya et al. [44] revealed that the blood samples of dementia patients displayed a significant increase in the levels of kynurenine and indoxyl sulfate, both of which are activating ligands of Ah receptor. These studies are backed up by the reports of robust changes in the micro-organism profiles in gut samples of AD patients as compared to those of healthy controls [45].

Many investigators have attempted to modulate the composition of microbiota to clarify the role of the microbiome in the progression of AD pathology [3, 4, 46, 47]. Germ-free mice and fecal microbiota transplantation as well as treatments with antibiotics, prebiotics, and probiotics have been exploited to reveal how the microbiota alter cerebral functions, especially in promoting the pathology underpinning AD. For instance, Harach et al. [46] reported that the germ-free transgenic APP/PS1 mice, i.e., animals without any gut microbiota, displayed a significantly reduced level of β-amyloid peptides and deposits in the brain as compared to the conventionally-raised APP/PS1 transgenic mice. Accordingly, there was a clear decrease in the level of cortical neuroinflammation in the germ-free transgenic AD mice. Interestingly, Harach et al. [46] also demonstrated that the transplantation of fecal microbiota from the aged APP/PS1 mice to the adult germ-free AD mice considerably increased the accumulation of cerebral β-amyloid peptides in these recipient mice. Kim et al. [47] reported that the repeated transfer of a healthy gut microbiota from wild-type mice to the transgenic AD mice (ADLP^APT^) significantly reduced both amyloid and tau pathology. This kind of fecal transplantation decreased the accumulation of β-amyloid deposits and tau protein tangles in the brain as well as improving the memory deficits of the ADLPAPT mice. Furthermore, the transfer of healthy microbiota into the transgenic AD mice ameliorated the intestinal and systemic inflammation and reduced their impaired gut permeability. Accordingly, Minter et al. [48] revealed that treatments with antibiotics strongly affected the composition of gut microbiota in the transgenic APP_SWE_/PS1_∆E9_ mice. Surprisingly, antibiotic treatments reduced the deposition of β-amyloid plaques and gliosis in transgenic AD mice. These studies indicate that the presence of inflammatory gut microbiota seems to promote AD pathology, whereas the transplantation of a healthy microbiota ameliorates intestinal inflammation and consequently delays the progression of AD pathology. However, the results of animal studies need to be confirmed in clinical human trials.

Given that AD pathology and neuropsychiatric disorders are associated with dysbiosis in gut microbiota, treatments with different compositions of probiotics have been explored in therapeutic trials. There are several systematic reviews of these interventions in human patients [49, 50] as well as in experiments with AD mice [51]. Briefly, it has been commonly observed that probiotic treatments are able to improve cognitive functions in both MCI and AD patients. This might be attributed to a decrease in the level of inflammation in both the gut and the brain. *Lactobacillus* and *Bifidobacteria* have been the most frequently administered probiotic genera in the therapeutic interventions. However, there are considerable differences between trials with respect to the composition of the probiotic supplementation as well as in the therapeutic outcome. Currently there is no consensus regarding the formulation of bacteria, optimal doses, or treatment schedules to achieve an optimal response with probiotics for AD therapy. Moreover, the molecular mechanisms behind the therapeutic responses evoked by probiotics to AD brains need to be clarified. There is an increasing number of studies which have examined whether prebiotic compounds can prevent or delay AD pathology [52, 53]. Prebiotics are dietary compounds which exert healthy responses in gut microbiota, e.g., nondigestible fibers are able to enhance the growth, survival, and activity of gut microbiota. There is clear epidemiological evidence that some diets are associated with cognitive impairment, whereas other diets can improve cognitive functions and memory [52]. For instance, it is known that the Mediterranean diet can reduce the age-related cognitive decline and reduce the risk for AD pathology [52, 54]. Given that the Mediterranean diet has a profound effect on the gut microbiota [55], it seems likely that its beneficial effects on dementia and AD pathology are mediated by alterations in the gut microbiota.

## Gut host-microbiota, tryptophan metabolites, and activation of AhR signaling

**Fig. 1:**
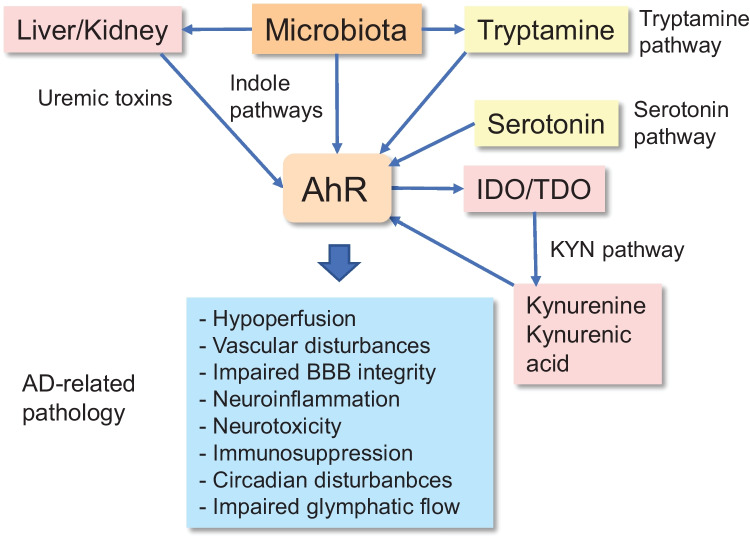
Gut host-microbiota-derived tryptophan metabolites activate AhR signaling in the brain via different pathways and induce pathological changes observed in AD pathology. Microbial bacteria secrete indole molecules which via circulation can activate AhR signaling in the brain. Liver and kidney process the secreted indole molecules into uremic toxins, e.g., indole sulfate. Uremic toxins accumulate in the blood during chronic kidney disease. There is a close interplay between the microbiota and the host cells of the gastrointestinal tract, stimulating the generation of serotonin by enterochromaffin cells. Some microbes can convert tryptophan into tryptamine which activates AhR signaling. Gut host-microbiota interplay, e.g., in inflammation, activates the IDO1/TDO-induced KYN pathway which generates novel agonists for the Ah receptor. Subsequently, AhR signaling stimulates the expression of IDO1 enzyme inducing a positive feedback loop. Below, the pathological effects induced by AhR signaling are listed, many of which are also observed in AD pathology (see the text). Abbreviations: AD, Alzheimer’s disease; AhR, aryl hydrocarbon receptor; BBB, blood–brain barrier; IDO1, indoleamine 2,3-dioxygenase, KYN, kynurenine; TDO, tryptophan 2,3-dioxygenase

The microbiota has a crucial role in the regulation of immune homeostasis not only in the intestine but also in the body via the circulation. There is clear evidence that the microbiota exploits tryptophan metabolites and SCFA for messenger functions and it seems that the Ah receptor is the major target of the tryptophan metabolites derived from intestinal microbiota [7, 56] (Fig. 1). In addition, it is known that SCFA stimulate the expression of AhR factors (see below). As discussed above, AhR signaling exerts beneficial and harmful properties, both during the developmental phase and later in life. Furthermore, as stated, its activation is associated with the development of many chronic diseases. For instance, AhR signaling is able to inhibit inflammatory conditions by activating anti-inflammatory and immunosuppressive responses, thus maintaining intestinal host-microbiota homeostasis. However, the presence of chronic inflammation in the gut stimulates the release of tryptophan metabolites and cytokines into the circulation inducing pathological alterations, e.g., in the brain and the kidney. Overall, the well-being of gut host-microbiota is dependent on dietary components, either directly or through the microbiota-generated metabolites, thus having an impact on human health.

## Gut host-microbiota produces tryptophan metabolites associated with AD pathology

**Fig. 2:**
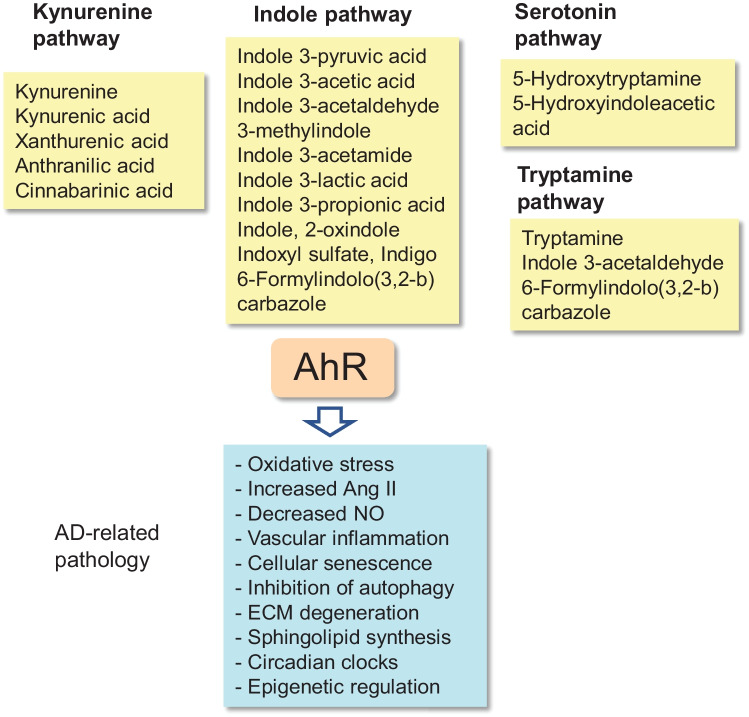
AhR agonists generated via different tryptophan metabolic pathways. The AhR-activating metabolites produced by the kynurenine, indole, serotonin, and tryptamine pathways have been compiled. The pathological responses which also exist in AD pathology are displayed underneath. The detailed reactions have been explained in the text

The majority of dietary l-tryptophan liberated in the intestine is transferred through the epithelium into circulation; about 10–20% of l-tryptophan is metabolized by cells of the intestinal epithelium and microbiota in gut lumen [6]. There are four major routes through which the gut host-microbiota can catabolize dietary l-tryptophan to active metabolites, i.e., (i) indole, (ii) kynurenine (KYN), (iii) serotonin, and (iv) tryptamine pathways [7, 37, 57, 58]. Several bacteria in gut microbiota, e.g., *Lactobacillus* ssp., *Bifidobacterium* spp., and *Peptostreptococcus russellii*, contain a tryptophanase enzyme, which can convert l-tryptophan into indole 3-pyruvic acid [7, 37]. This rate limiting compound can be processed to numerous indole derivatives in gut microbiota and also in human liver and kidney. For instance, indole 3-acetaldehyde, indole 3-acetic acid, 3-methylindole, and indole 3-aldehyde are important down-stream derivatives [7] (Fig. 2). Indole 3-pyruvic acid can also be deaminated into indole 3-propionic acid which is claimed to be a neuroprotectant [59]. Hendrikx and Schnabl [60] have described in detail the indole chemical pathways in intestinal bacteria. In fact, indole is an important intercellular messenger in the communication between bacterial species [61]. However, many indole molecules are potent activators of AhR signaling (Fig. 2) which can exert many harmful effects on human health [37]. For instance, the microbiota-generated indole molecules can be converted in human liver into indoxyl sulfate which exerts detrimental effects especially on vascular homeostasis and AD pathology (Fig. 1). The role of indoxyl sulfate in AD pathology has been addressed below in the Section on Indoxyl sulfate and AD pathology.

The 6-formylindolo[3,2-b]carbazole (FICZ) is another interesting indole derivative of l-tryptophan [62, 63]. There exist several pathways which process l-tryptophan to FICZ, either non-enzymatic oxidative pathways driven by UVB and H_2_O_2_ or enzymatic pathways from indole 3-pyruvic acid or indole 3-acetaldehyde [62, 64]. FICZ is a strong inducer of AhR signaling in the gut generated by both microbiota and the intestinal host tissues [63]. FICZ is catabolized by CYP1A1/1A2 and CYP1B1, transcriptional targets of AhR signaling [65, 66]. This generates an interesting autoregulatory feedback loop where the activity of CYP enzymes controls the level of FICZ and thus the activation of AhR signaling [62, 66]. FICZ has a number of physiological functions in the differentiation of stem cells and immune cells, e.g., in gut immunity [62, 63]. FICZ can also improve mouse hippocampal neurogenesis as well as memory and learning properties [67]. Given that AhR signaling exhibits clear evidence for antagonistic pleiotropy [9], the FICZ-induced AhR signaling also stimulates oxidative stress and pro-inflammatory responses [68]. Moreover, there are indications that FICZ is involved in the photoaging process of the skin [69, 70].

There is a close interplay between the host’s intestinal cells and the microbiota in the gastrointestinal tract in the maintenance of intestine homeostasis [6, 37]. In the cells lining the intestinal wall, indoleamine 2,3-dioxygenase 1 (IDO1) and tryptophan 2,3-dioxygenase (TDO) catabolize l-tryptophan first into KYN and consequently into several other metabolites, e.g., kynurenic acid (KYNA), 3-hydroxykynurenine (3-HK), cinnabarinic acid (CIN), quinolinic acid (QUIN), and picolinic acid (PIC) [57, 71]. It is known that the metabolites of the KYN pathway can trigger either neurotoxic or neuroprotective effects. For instance, 3-HK and QUIN are neurotoxic metabolites, whereas KYNA and PIC are neuroprotective [72, 73]. Accordingly, some of the metabolites enhance inflammatory responses, while some are anti-inflammatory and immunosuppressive. For instance, KYN and KYNA stimulate AhR signaling and subsequently they enhance anti-inflammatory/immunosuppressive responses [74, 75]. On the contrary, 3-HK and QUIN are agonists for N-methyl-d-aspartate (NMDA) receptors and they can trigger excitotoxic and inflammatory responses which are evident in many neurodegenerative diseases [74, 75]. Moreover, the presence of inflammatory cytokines can stimulate the expression of IDO1 and thus enhance the activity of the KYN pathway [76] and subsequently stimulate AhR signaling There is clear evidence that the aging process is associated with an increase in the catabolism of tryptophan and the activation of KYN signaling, e.g., in rat brain [77, 78]. Accordingly, several studies on AD pathology have revealed that the catabolism of tryptophan is elevated, and consequently, the levels of KYN and QUIN are increased in plasma and cerebrospinal fluid (CSF) [42, 79].

The gut enterochromaffin cells can convert dietary l-tryptophan into serotonin [7, 57]. However, it seems that gut microbiota can regulate both the synthesis and release of serotonin from gut enterochromaffin cells [80, 81]. For instance, Reigstad et al. [80] demonstrated that SCFAs produced by microbiota promoted serotonin production in human enterochromaffin cells. Tryptophan hydroxylase 1 (TPH1), the rate-limiting enzyme in the synthesis of serotonin, catabolizes the conversion of l-tryptophan into 5-hydroxytryptophan which subsequently is decarboxylated into 5-hydroxytryptamine (5-HT), i.e., serotonin. Consequently, 5-HT can be converted into melatonin which controls many properties of intestinal microbiota, e.g., oxidative stress and inflammation [82]. 5-HT can also be metabolized into 5-hydroxyindole acetic acid (5-HIAA) [7]. Both 5-HT and 5-HIAA are agonists for AhR signaling (Fig. 2). Gut host-microbiota has a crucial role in the total biosynthesis of serotonin since over 90% of the serotonin present in the body is synthetized in the gut [81]. Spore-forming bacteria, e.g., *Clostridium ramosum*, are major enhancers of serotonin production in enterochromaffin cells [83]. It is known that serotonin can attenuate the severity of inflammation and allergic diseases in the intestine. Serotonin also regulates gastrointestinal motility and platelet function in mice [81]. In the brain, the TPH2 isoform is the dominant enzyme in the synthesis of serotonin. Currently, it is not known whether serotonin generated by the gut can directly affect the serotonergic transmission in the brain since the BBB is impermeable to biogenic amines [84]. Interestingly, there is abundant evidence that serotonin can regulate the function of immune cells and thus control inflammation and immunity [85]. There is clear evidence that the serum levels of serotonin decline significantly in patients with AD [43]. This might indicate that serotonin production in the gut is reduced in AD patients. It is known that there exists a regulatory co-operation between the serotonin, kynurenine, and indole pathways [86]. However, it is not known whether changes in the bacterial composition of microbiota in AD patients are able to modulate the generation of different types of tryptophan metabolites.

The tryptamine pathway is the fourth route to metabolize dietary l-tryptophan exploited by gut microbiota [37, 87]. Some commensal bacteria, e.g., *Ruminococcus gnavus* and *Clostridium sporogenes*, possess a tryptophan decarboxylase enzyme which catabolize dietary l-tryptophan into tryptamine and subsequently tryptamine can be converted to 2-dimethyltryptamine. Monoamine oxidases A and B can metabolize tryptamine to indole 3-acetaldehyde (Fig. 2). In contrast to serotonin, tryptamine and 2-dimethyltryptamine are able to penetrate through the BBB and thus affect cerebral functions. Tryptamine exerts many functions in the maintenance of gut homeostasis, e.g., it regulates serotonin metabolism and immune functions. However, tryptamine and 2-dimethyltryptamine are hallucinogenic compounds [87] and these tryptophan metabolites have been implicated in various neuropsychiatric disorders [88]. Paley et al. [89] demonstrated that tryptamine inhibited tryptophanyl-tRNA synthetase (TrpRS) which is known to accumulate into senile plaques in the brains of AD patients. They reported that tryptamine exposure, both in human cell lines and mouse models, induced a type of neurodegeneration involving the formation of neurofibrillary tangles. Given that tryptamine is a potent agonist of the Ah receptor (Fig. 2), Dopkins et al. [90] reported that mice administered tryptamine displayed ameliorated symptoms of experimental autoimmune encephalomyelitis (EAE) through the activation of AhR signaling. For instance, tryptamine exposure increased the number of immunosuppressive FoxP3-positive Tregs in mouse EAE brain. This implies that tryptamine generation was able to inhibit inflammatory responses via AhR signaling.

## Tryptophan metabolites are activators of AhR signaling

Originally, it was thought that the Ah receptor is a chemical sensor against environmental contaminants, such as toxic TCDD and PAH compounds, inducing their detoxification [15, 18]. Subsequently, it was observed that not only xenobiotic chemicals but also many products of the gut microbiota were agonists for Ah receptor. In particular, tryptophan metabolites are well-known inducers of AhR signaling [56]. Tryptophan metabolites are not only crucial regulators of intestinal homeostasis and mucosal immunity but due to their presence in the circulation, they can regulate the functions of vascular system and peripheral tissues, such as the brain. It is known that the bacterial composition has a crucial role in the generation of different tryptophan metabolites in the gut. For instance, Hubbart et al. [91] have described the molecular structures and the synthesis routes of microbial indole derivatives. Moreover, kidney and liver can convert the indole molecules delivered by circulation into indoxyl sulfate and some other indole derivatives [91]. Several investigators have compared the potency of different indole derivatives to act as agonists of the Ah receptor in isolated AhR preparations and in various cell types [56, 92, 93]. Most of the indole derivatives are agonists for the Ah receptor although their affinity, potency, and efficacy in the AhR-CYP1A1 pathway vary extensively [93]. However, indole derivatives are clearly less potent agonists than environmental chemicals, such as TCDD and PAH. Indole, 3-methylindole, indole 3-pyruvate, and indole 3-acetamide were the most potent indoles to elicit nuclear translocation of AhR protein and the binding of the AhR complex to the promoter of *CYP1A1* gene in human intestinal LS180 and HT-29 carcinoma cells [93]. Indole derivatives induced similar responses in primary human hepatocytes. Interestingly, Jin et al. [94] demonstrated that indoxyl sulfate, an indole derivative produced by kidney and liver, was clearly a more potent inducer of AhR signaling than the indole molecule itself in the AhR-mediated induction of CYP1A1 expression in human CaCo-2 cells. This may explain why indoxyl sulfate has many toxic effects related to Alzheimer’s pathology (see below). The major indole molecules characterized as agonists for the Ah receptor have been listed in Fig. 2.

There is a close cooperation between the indole and kynurenine pathways in the control of immunity and homeostasis in the gut. For instance, the indole derivatives released from microbiota can stimulate AhR signaling in the host cells of gastrointestinal tract, known to be enriched of AhR proteins (Human Protein Atlas). It is known that AhR signaling stimulates the transcription of the *IDO1* gene [95]. Subsequently, the IDO1 enzyme activates the KYN pathway which generates a number of KYN metabolites (see above). These derivatives can be released into the intestinal lumen and the circulation. Interestingly, Mezrich et al. [75] demonstrated that KYN but not 3-HK or QUIN were able to activate AhR signaling in mouse CD4T, DC, and hepatoma cells. They also revealed that KYN/AhR signaling stimulated the differentiation of immunosuppressive regulatory T cells (Treg) which have an important role in the suppression of inflammatory responses, in the gut as well as in other parts of the body. DiNatale et al. [74] reported that KYNA also stimulated AhR signaling in mouse HepG2 and human primary hepatocytes. Subsequently, it has been observed that cinnabarinic acid and xanthurenic acid, two downstream metabolites of the KYN pathway, are also potent agonists for AhR signaling [7] (Fig. 2). The AhR-IDO1-KYN-AhR pathway establishes an interesting feedback loop which can be enhanced by the indoles generated by microbiota. Interestingly, the tryptophan metabolites of the serotonin and tryptamine pathways can also stimulate AhR signaling [92, 94, 96]. It seems that tryptophan metabolism activated in gut host-microbiota is an effective endogenous source of AhR agonists which are potent immunomodulators, both in the gut and elsewhere in the body.

## Gut inflammation enhances production of kynurenine and its metabolites

The activation of KYN pathway is a common hallmark of chronic inflammatory conditions in many gastrointestinal diseases, e.g., in inflammatory bowel disease (IBD) and ulcerative colitis [97, 98]. IDO1 is a rate-limiting enzyme for the KYN pathway and it is abundantly expressed in the gastrointestinal tract (Human Protein Atlas). A significant increase in serum levels of KYN/KYNA has been observed in IBD [99], Crohn’s disease [100], and ulcerative colitis [98]. Concurrently, there were an elevation of IDO1 activity in human colonic mucosa. It is known that many pro-inflammatory cytokines stimulate the expression of IDO1 through the activation of NF-κB and JAK-STAT3 signaling [101]. For example, some inflammatory gut diseases are associated with an increase in the serum levels of many pro-inflammatory cytokines which can act in remote tissues, such as in the brain and kidney. However, the metabolites of KYN pathway can exert both beneficial and detrimental effects on vascular endothelium and target tissues. It is known that KYN can easily penetrate through the BBB and gain access into the brain [102]. Many epidemiological studies have revealed that IBD is associated with a higher dementia risk [103] as well as a risk for AD and other neurodegenerative diseases [104, 105]. Owe-Young et al. [106] demonstrated that endothelial cells and pericytes, cells of the BBB, contained the enzymes which are able to process KYN into neurotoxic compounds, such as QUIN and 3-HK. Interestingly, Zhang et al. [107] demonstrated that KYN treatment of mouse primary astrocytes, also a partner cell in the BBB, robustly upregulated the expression of Nod-like receptor protein 2 (NLRP2). The activation of NLRP2 inflammasomes stimulated the secretion of IL-1β and IL-18. Zhang et al. [107] also reported that an intraperitoneal injection of KYN stimulated astrocytic NLRP2 inflammasomes in mouse hippocampus, inducing a depression-like behavior. The depletion of astrocytic NLRP2 abolished these depressive symptoms. These results indicate that KYN can penetrate through the BBB and trigger neuroinflammation. The potential role of microbiota-derived KYN and its metabolites in the regulation of brain physiology and pathology has been recently reviewed in detail elsewhere [108].

## Indoxyl sulfate and AD pathology

Indole molecules secreted from the gut can be metabolized in the liver to indoxyl sulfate (Fig. 1). The hepatic microsomal enzyme CYP2E1 hydroxylates indole into indoxyl molecule which is subsequently converted into indoxyl sulfate by the sulfotransferase SULT1A1 [109, 110]. The clearance of indoxyl sulfate from the circulation through the kidney was clearly reduced in chronic kidney disease (CKD) leading to an accumulation of indoxyl sulfate in the blood, i.e., it is a uremic toxin [111, 112]. Indole 3-acetic acid and 2-oxindole are also uremic toxins because their levels in serum were significantly increased in CKD [113, 114]. Interestingly, indoxyl sulfate was clearly a more potent agonist molecule than indole itself, i.e., its hydroxylation/sulfation robustly increased its activity as an AhR agonist in human CaCo-2 intestinal cells [94]. Walker et al. [115] demonstrated that an experimental increase in indoxyl sulfate in the blood of transgenic DRE-LacZ mice significantly stimulated AhR signaling in kidney, liver, heart, and the microvasculature of the cerebral cortex. They also revealed that the AhR activity of these tissues was significantly increased in the mouse models of CKD and acute kidney injury (AKI). Histochemical assays revealed that an increase in the serum level of indoxyl sulfate stimulated AhR signaling in vascular tissues as well as in hepatocytes and cardiac myocytes. This indicates that indoxyl sulfate is able to stimulate the expression of AhR protein not only in blood vessels but also in the cells of many organs. It is commonly believed that indole molecules secreted from the gut are only metabolized into indoxyl sulfate in the liver because the expression of CYP2E1 protein is enriched in the liver (Human Protein Atlas). However, there are studies indicating that CYP2E1 enzyme is expressed throughout the human gastrointestinal tract [116]. Moreover, sulfotransferases, including SULT1A1, are highly expressed in all parts of human gastrointestinal tract, at an even higher level than in the liver [117]. In the colon, SULT1A1 protein was expressed in mature enterocytes and endothelial cells. It is most probable that indole molecules can be converted into toxic indoxyl sulfate already in the gut from where they will be secreted into the circulation.

There are studies indicating that the serum level of indoxyl sulfate is significantly increased in neuropsychiatric disorders [118] and dementia [44]. Moreover, it is known that AD pathology is associated with CKD in humans [119] and in transgenic AD mice [120]. Many studies have revealed that dysbiosis of gut microbiota enhances the pathogenesis of CKD [112, 121]. It seems that an increase in the serum level of indoxyl sulfate in CKD (see above) could enhance the pathogenesis of AD. It is known that indoxyl sulfate can disrupt the integrity of BBB via the activation of AhR signaling in experimental CKD models of rats and mice and subsequently induce cognitive impairment [122].

It is known that indoxyl sulfate can trigger several toxic effects, especially on the vascular tissues. Indoxyl sulfate is transported into cells via the organic anion transporters (OAT) [123], e.g., OAT3 is extensively expressed in the BBB [124]. There is convincing evidence that an increase in the level of serum indoxyl sulfate can induce vascular calcification and promote endothelial senescence [125, 126]. Indoxyl sulfate is able to disturb the functions of endothelium in several ways, e.g., (i) it increases vascular permeability [127], (ii) it impairs endothelium-dependent vasorelaxation [128], (iii) it inhibits nitric oxide (NO) production and impairs vasodilation [129], (iv) it stimulates leukocyte adhesion to endothelial cells [130], (v) it activates the expression of tissue factor inducing platelet activation and thrombosis [131], (vi) it suppresses the angiogenesis mediated by endothelial progenitor cells [132], and (vii) it induces cellular senescence of endothelial cells [125]. Moreover, indoxyl sulfate has been demonstrated to promote the proliferation of vascular smooth muscle cells [133] and induce vascular inflammation [134]. Indoxyl sulfate also affects hemodynamics, e.g., an intravenous injection of indoxyl sulfate increased both heart rate and arterial blood pressure in rats [135]. There are reports indicating that indoxyl sulfate was able to stimulate the renin-angiotensin system, e.g., it activated the renin–angiotensin–aldosterone system in mouse kidney inducing kidney fibrosis [136]. Sun et al. [137] demonstrated that chronic intraperitoneal administration of indoxyl sulfate into mice increased its level in blood, prefrontal cortex, and cerebrospinal fluid (CSF). Indoxyl sulfate treatment decreased some parameters of neuronal survival, e.g., the levels of neurotrophins, whereas it increased oxidative stress, and induced neuroinflammation. These are also common markers of AD pathology. Interestingly, it seems that the harmful effects of indoxyl sulfate on the vascular system and tissue homeostasis are caused by the activation of AhR signaling (see details below).

## Short-chain fatty acids (SCFA) enhance AhR signaling

The gut microbiota generates several SCFA, especially acetate, butyrate, and propionate, which have some beneficial effects on gastrointestinal health but they can affect via circulation the functions of the brain and other tissues [138]. Commonly, SCFA activate the free fatty acid receptors 2/3 (FFAR2/3), present also in the human BBB [139, 140]. However, there is convincing evidence that SCFA can activate AhR signaling without the activation of FFAR2/3 signaling. It is known that SCFA, especially butyrate, are inhibitors of histone deacetylases (HDACs) which are potent regulators of gene expression. Garrison and Denison [141] reported that butyrate strongly increased (by eightfold) the activity of the AhR promoter in mouse Hepa-1 cells. Recently, Modoux et al. [142] demonstrated that butyrate treatment robustly increased the activity of AhR signaling in human HepG2 cells. They also revealed that butyrate treatment clearly enhanced the activation of AhR signaling induced by the FICZ ligand, a tryptophan metabolite (see above). Modoux et al. [142] also reported that butyrate and other SCFA were not ligands for the Ah receptor, i.e., these compounds did not bind to the AhR protein. This means that SCFA activated AhR signaling through the inhibition of HDACs, thus transactivating the *AhR* gene. Korecka et al. [143] demonstrated that SCFA exposure increased AhR signaling and its target gene expression in mouse intestine and liver. Accordingly, they revealed that AhR signaling was able to control the composition of the microbiota in mouse intestine. Jin et al. [144] demonstrated that SCFA enhanced histone acetylation which increased the recruitment of the Ah receptor to the promoter of the *CYP1A1* gene in mouse and human colonic cell lines. These studies indicated that the microbiota-derived SCFA are able to stimulate histone acetylation which leads to an opening of the DNA-binding sites for the ligand-activated Ah receptors inducing the transactivation of target genes. This strongly augments the capability of tryptophan metabolites to induce AhR-dependent responses.

Currently, there are inconsistent results on the role of SCFA in AD pathogenesis. It is known that many SCFA, especially butyrate, are strongly anti-inflammatory in rat primary microglia and hippocampal slice cultures [145]. Jiang et al. [146] reported that administration of sodium butyrate relieved neuroinflammation in transgenic 5xFAD mice and ameliorated many markers of AD pathology. In addition, Fernando et al. [147] revealed that chronic administration of sodium butyrate at an early stage of AD reduced the deposition of β-amyloid and improved cognitive memory in 5xFAD mice. On the other hand, exploiting germ-free technology and SCFA supplementation in transgenic AD mice, Colombo et al. [148] convincingly demonstrated that the microbiota-derived SCFA promoted AD pathology, e.g., by increasing the activation of microglia and β-amyloid deposition. Moreover, Marizzoni et al. [149] reported results indicating that the levels of serum SCFA, especially acetate and valerate but not propionate, as well as some cytokines, e.g., IL-1β, correlated with the markers of endothelial dysfunction and the β-amyloid load in AD patients. These results emphasize the role of endothelial dysfunction in the amyloid pathology induced by SCFA and inflammatory cytokines.

## Impact of gut-derived AhR agonists in AD pathogenesis

There is an extensive literature indicating that a disruption of the BBB has a crucial role in the development of AD, possibly even driving AD pathogenesis. Cerebral amyloid angiopathy (CAA) is a hallmark of AD pathology indicating that vascular dysfunction has an important role in the pathogenesis of AD. Interestingly, the expression of AhR protein is robustly enriched in the BBB, suggesting that tryptophan metabolites can affect the function of microvessels, especially that of the BBB (see below). AhR signaling can trigger many harmful effects which means that AhR agonists derived from gut host-microbiota can disturb microvessels, thus leading to a loss of BBB integrity. For instance, AhR signaling is able to induce hypoperfusion in local brain regions via stimulating the renin-angiotensin system or inhibiting NO production (see below). Hypoperfusion leads to many detrimental secondary injuries enhancing AD pathogenesis. AhR signaling also disturbs the regulation of circadian rhythms in the brain increasing BBB permeability and probably impairing glymphatic functions.

## Expression of AhR factor is enriched in the BBB and regulates its integrity

The BBB consists of the endothelial cells around the capillary lumen, pericytes embedded in the basement membrane, and astrocytes supporting the capillary with end-feet [150]. Endothelial cells are attached to each other with tight and adherens junctions in cerebral microvessels. The BBB is selectively permeable preventing compounds circulating in the bloodstream from passing to the extracellular space of the brain. There are two mechanisms through which compounds can pass through the BBB, i.e., passive diffusion of small non-polar molecules and active transport for certain compounds via different mechanisms. Cerebral blood flow (CBF) is controlled by the neurovascular unit composed of endothelium, astrocytes, pericytes, and vascular smooth muscle cells, a process called neurovascular coupling [151]. Interestingly, the expression of the Ah receptors is highly enriched in the cells of the BBB [152–155]. Given that AhR protein is abundantly expressed in many barrier organs [24], it seems that Ah receptor also possesses an important role as an environmental sensor in the BBB. For example, the activation of AhR signaling with TCDD strongly increased the expression of AhR-target genes, i.e., CYP1A1 and CYP1B1 in mouse endothelial cells and astrocytes [152] as well as P-glycoprotein, an ATP-driven transporter, in the BBB [153]. There is convincing evidence that disturbances in the integrity of the BBB promote AD pathogenesis [12, 13] and inducing cognitive impairment [156]. Currently, the mechanisms causing a loss of BBB integrity in AD pathogenesis need to be clarified.

There are several studies indicating that a loss of BBB integrity is induced by an activation of AhR signaling. For instance, Bobot et al. [122] demonstrated that the overload of indoxyl sulfate in the bloodstream induced by a deficient function of mouse kidney (induced by either feeding an adenine-rich diet or partial nephrectomy) robustly activated AhR signaling which increased the permeability of BBB and evoked cognitive impairment. They also confirmed these results by demonstrating that the administration of indoxyl sulfate in drinking water was also able to induce BBB disruption along with signs of a cognitive impairment. Indoxyl sulfate exposure in AhR knockout mice did not increase BBB permeability or cause any cognitive impairment indicating that the activation of AhR signaling disturbed the integrity of the BBB. Ren et al. [155] revealed that the hyperglycemia-induced mouse intracerebral hemorrhage (ICH) significantly increased the expression of AhR protein in the BBB. AhR signaling augmented the expression of thrombospondin-1, TGF-β, and VEGF, whereas those of ZO-1 and claudin-5, common proteins of tight junctions, significantly decreased. Hyperglycemic-ICH increased BBB disruption and hematoma expansion. In contrast, the knockout of AhR factor in vivo with the CRISPR technique reduced the expression of Ah receptor, ameliorated BBB disruption, and improved neurobehavioral functions. Moreover, Maciel et al. [157] demonstrated that the treatment of human endothelial cells (EA.hy926) with indoxyl sulfate clearly decreased the expression of VE-cadherin protein, a tight junction protein, and reduced the cell-to-cell junctions of human endothelial cells. It is known that the expression of claudin-5 is significantly down-regulated in the frontal cortex of patients with Alzheimer’s disease and vascular dementia [158]. The mechanism underpinning the AhR-mediated loss of tight junctions in the BBB still needs to be clarified. Chang et al. [159] demonstrated that the activation of AhR/RhoA signaling elicited a degradation of β-catenin protein in mouse cerebral endothelial cell cultures. The down-regulation of β-catenin protein was induced by its phosphorylation through PKCδ and GSK3β. Interestingly, they revealed that treatment of mice with simvastatin and pravastatin inhibited the degradation of β-catenin by inhibiting RhoA, and consequently, these treatments prevented the AhR-mediated disruption of brain vascular integrity. These are only some examples and currently it is known whether AhR signaling has other mechanisms through which it can affect the integrity of brain microvessels.

## AhR signaling controls blood flow in brain via renin-angiotensin system

Many neuroimaging studies on AD patients have revealed that cerebral blood flow (CBF) is clearly reduced in the regions which are vulnerable to AD pathology [160, 161]. Chronic hypertension causes vascular injuries which might induce a local hypoperfusion and lead to AD pathology. There is convincing evidence that changes in AhR signaling are associated with an increase in systemic arterial hypertension [162, 163]. Interestingly, there are studies indicating that dysbiosis of gut microbiota can increase the arterial blood pressure [164]. Currently, it is known that several mechanisms regulate the AhR-driven hemodynamics both at the systemic level and in the brain (see below). The renin-angiotensin system (RAS) has a crucial role in the control of local and systemic blood pressure [165]. It is known that dysbiosis of microbiota affects the function of RAS at both at the systemic and local levels [166]. In the brain, there exists a local RAS, distinct from the systemic endocrine renin–angiotensin–aldosterone system (RAAS) [167]. This brain-specific RAS has an important role in the control of brain hemodynamics as it is able to induce either vascular constriction or dilatation. It has been shown that all of the components of the RAS are expressed in the brain, although renin, normally released from liver, might be produced by a prorenin (PR)/prorenin receptor (PRR) interaction in brain [167]. Yisireyili et al. [133] demonstrated that indoxyl sulfate induced the expression of PR and PRR proteins in rat aorta and human aortic smooth muscle. They also reported that the increased expression was dependent on ROS, OAT3, Ah receptor, and the AhR-mediated activation of NF-κB signaling, i.e., the RAS system was being driven by AhR signaling in brain microvessels.

The renin/PR proteins convert the angiotensinogen protein into angiotensin I (Ang I) which is subsequently cleaved into angiotensin II (Ang II) by angiotensin converting enzyme (ACE). Exposure to indoxyl sulfate significantly increased the expression of angiotensinogen protein in human proximal tubular cells (HK-2) and rat renal cortex, thus leading to RAS activation and the generation of Ang II peptides [168]. In the brain, Ang II increases the production of vasopressin and induces vasoconstriction by activating the angiotensin II type 1 receptor (AT1R) [169–171]. Zhang et al. [172] demonstrated in mice that an infusion of Ang II for two weeks induced cerebral microvascular inflammation and increased the permeability of mouse BBB. The Ang II-mediated hypertension promoted ROS production and enhanced leukocyte adhesion to the brain vasculature. Vital et al. [170] reported that AT1R was responsible to the Ang II-induced vascular adhesion of leukocytes and platelets as well as the increased permeability of the BBB. Interestingly, Agbor et al. [173] revealed that the knockout of endothelial cell-specific AhR in mice displayed a hypotensive phenotype and the responsiveness of these mice to Ang II treatment was clearly declined. They also reported that the expression of AT1R protein in the aorta was significantly reduced in the knockout mice indicating that AhR signaling might control the expression level of the AT1R protein. There are studies indicating that certain AhR agonists, such as indoxyl sulfate and hexachlorobenzene (HCB) induce hypertension and vascular dysfunctions in mice [135, 174]. Sun et al. [136] demonstrated that exposure to indoxyl sulfate activated the intrarenal RAAS involving an increase in the expression of renin, angiotensinogen, and AT1R proteins. They revealed that the indoxyl sulfate-stimulated RAAS was mediated by an activation of TGF-β signaling. Ongali et al. [175] reported that in mouse brain, the overexpression of TGF-β signaling induced cerebrovascular dysfunctions and astrogliosis through the activation of AT1R signaling. Moreover, Wyss-Coray et al. [176] demonstrated that overproduction of TGF-β factor promoted a microvascular degeneration reminiscent of AD pathology. It is known that there is a complex, context-dependent regulation between AhR and TGF-β signaling pathways. For instance, Ren et al. [155] revealed that the AhR signaling induced by hyperglycemia in mice stimulated the thrombospondin/TGF-β pathway and impaired the function of the BBB.

It is known that Ang II peptide can also activate AT2R and Mas receptor (MasR) which display counteracting effects to the AT1R, i.e., they promote vasodilation, decrease blood pressure, and stimulate responses against inflammation and fibrosis [171]. Post-mortem studies on AD brains have revealed that the expression levels of AhR, Ang II, and AT1R proteins were significantly increased, whereas that of MasR protein was clearly down-regulated in hippocampus samples [10, 177]. The expression level of AT2R protein was low and no difference was observed between the AD and non-AD groups. In addition, there was a lack of statistically significant changes in ACE-1 and ACE-2 protein levels in AD brains [177]. These studies indicate that both the expression of Ah receptor and the activation level of the AT1R/RAS signaling might be significantly increased in AD brains. It is known that an enhanced signaling through the Ang II/AT1R pathway activates NADPH-oxidase complex in rat brain, triggering the polarization of microglia into the pro-inflammatory M1 phenotype and leading subsequently to a local neurodegeneration [178]. There are active drug development programs to find whether the inhibitors of ACE-2 and AT1R could inhibit the cognitive impairment induced by the aging process and AD. Currently, most of the studies have revealed a mild improvement in the preservation of memory although it is not clear what is the best medication. With respect to AhR signaling, it is not known whether microbiota-derived metabolites induce the RAS-dependent constriction of microvessels and cause local hypoperfusion or whether AhR antagonists would be of any benefit in combatting AD pathogenesis.

## AhR signaling induces vascular disturbances

There is a reciprocal regulation between Ang II and nitric oxide (NO) in the regulation of blood pressure, i.e., Ang II is a major vasoconstrictor of vessels, whereas endothelial NO synthase (eNOS) is an important vasodilator [179]. eNOS produces NO which triggers relaxation of smooth muscle cells enhancing vasodilatation of cerebrovascular vessels. There is convincing evidence that AhR signaling inactivates the eNOS enzyme and inhibits NO production, thus impairing CBF [32, 180]. ROS compounds, especially the superoxide radical, react with NO generating the peroxynitrite radical which exerts many pathological effects [181]. AhR signaling is a potent inducer of ROS production, e.g., by stimulating the expression of the components of NADPH oxidase [182]. For instance, Nakagawa et al. [183] demonstrated that AhR signaling stimulated ROS production via the activation of NADPH oxidase and reducing NO generation in rat aorta. Interestingly, many AhR agonists, e.g., indoxyl sulfate and 3-methylcholanthrene, inactivated NO production in mouse and human endothelial cells [180, 183]. Recently, Picon-Pages et al. [184] have reviewed the major beneficial and detrimental effects of NO in the brain. For instance, NO affects the functions of many other proteins via nitrosylation, nitrosation, and nitration reactions. Not only is nitrosative stress a harmful state but also a deficiency of NO is detrimental. There is compelling evidence that a shortage of NO leads to vascular stiffness and calcification as well as promoting the pathogenesis of atherosclerosis [185, 186]. Toda and Okamura [187] postulated that the NO-induced endothelial dysfunction could trigger local cerebral hypoperfusion which aggravated AD pathogenesis.

As described above, indoxyl sulfate has many detrimental vascular effects, especially on the functions of the BBB. The indoxyl sulfate-induced AhR signaling stimulates ROS production which induces harmful responses not only via the RAS and NO pathways (see above) but oxidative stress can also trigger inflammatory changes and cellular senescence in endothelial cells [188, 189]. It is known that the activation of AhR signaling is a major inducer of oxidative stress [190]. Consequently, ROS compounds can stimulate NF-κB signaling, a major inflammatory inducer, and several other signaling pathways promoting cellular senescence [191]. Moreover, ROS compounds enhance vascular calcification and atherosclerosis. Masai et al. [192] demonstrated that the indoxyl sulfate-activated NADPH/NF-κB signaling in HUVEC cells stimulated the expression of monocyte chemoattractant protein-1 (MCP-1), an important enhancer of vascular inflammation. Cerebral amyloid angiopathy (CAA) is associated with increased oxidative stress and inflammation in AD pathology [193]. Sun et al. [194] reported that replicative senescence in human brain microvascular endothelial cells (BMEC) impaired the non-amyloidogenic APP processing, whereas the BACE1-mediated amyloidogenic APP processing was robustly increased. Increased amyloidogenic processing in senescent BMECs could enhance the formation of CAA in AD patients. Interestingly, AhR signaling and inflammatory cytokines are effective inducers of the activation of IDO1 enhancing the generation of KYN metabolites, many of which are AhR agonists [23, 76]. It seems that there exists a positive feedback loop in microvessels induced by the microbiota-derived AhR agonists disturbing cerebrovascular homeostasis.

## AhR signaling induces pathological responses associated with AD pathology

Given that AhR signaling is driving antagonistic pleiotropy (see above), the activation of AhR signaling induces diverse cellular stresses, such as oxidative stress which promotes the aging process [9]. It has been proposed that oxidative stress plays a significant role in the pathogenesis of AD [195]. It seems plausible that ROS production induced by AhR signaling might trigger inflammatory responses in AD brains, especially in microvessels. AhR signaling can also disturb energy metabolic homeostasis of cells since the activation of Ah receptor stimulates the expression of TiPARP (PARP7) which depletes NAD^+^ storage and triggers protein mono-ADP-ribosylation [196]. A deficiency of NAD^+^ impacts on several hallmarks of AD pathology, e.g., mitochondrial dysfunction, redox impairments, compromised autophagy, and disturbances in chromatin and epigenetic regulation (Wang et al., 2021). There are also clinical and animal experiments indicating that the exposure to NAD^+^ precursors has positive effects on dementia and AD pathology [197]. Several neurodegenerative diseases, e.g., AD pathology, are associated with disturbances in the sphingolipid metabolism, which is especially enriched in the brain [198]. Interestingly, Majumder et al. [199] demonstrated that AhR signaling upregulated the expression of serine palmitoyltransferase small subunit A (SPTSSA) in the SPT complex, the rate limiting enzyme of sphingolipid synthesis. Accordingly, Wang et al. [200] revealed that AhR signaling inhibited the expression of human sphingosine-1-phosphate lyase (S1PL), the major enzyme involved in the degradation of S1P. This means that the activation of AhR signaling not only stimulates the synthesis of sphingolipids but furthermore it prevents their degradation. It is known that sphingolipids control many crucial functions in AD and other neurodegenerative diseases [198].

AhR signaling also controls a wide variety of cellular responses which enhance pathological responses not only with aging but also in many diseases, such as AD. For instance, AhR signaling inhibits autophagic degradation [29, 30] which represents a positive effect during the growth phase but later in the life it disturbs cellular proteostasis. It is known that autophagic degradation in neurons is impaired in AD pathology affecting not only the cleansing of neurons but also the processing of APP [201, 202]. Autophagy also alleviates the hypoxia-induced BBB injury maintaining the integrity of the BBB [203]. Cellular senescence is another state which can be triggered by AhR signaling [30, 125, 204]. For instance, Wan et al. [204] reported that TCDD, a strong agonist of the AhR, induced cellular senescence of human and rat neuronal cells by increasing ROS production. AhR signaling also induced cellular senescence mediated by indoxyl sulfate treatment in human endothelial HUVEC cells [125]. It seems that cellular senescence is not the primary cause of AD but nonetheless the appearance of senescent astrocytes can promote AD pathology [205]. Moreover, AhR signaling can induce the inflammatory state, and consequently, the activation of NF-κB signaling stimulates the expression of AhR protein because the promoter of the human *AhR* gene is under the transactivation of NF-κB factor [206]. NF-κB signaling also stimulates the expression of IDO1 enzyme which activates AhR signaling through the production of KYN metabolites. The inflammation-induced, probably also the inflammaging-induced, activation of AhR signaling stimulates the immunosuppressive differentiation of several immune cell types, e.g., M2 macrophages (Mreg) [207] and regulatory T cells (Treg) [208]. For instance, the activation of AhR signaling induced the expression of Forkhead box 3 protein (FoxP3) which is the master regulator of immunosuppressive Tregs [208]. Immunosuppression is one of the hallmarks of AD pathology, i.e., microglial cells are hyporesponsive and lose the ability to dispose β-amyloid deposits [209, 210]. Interestingly, the overexpression of TGF-β, a major immunosuppressive cytokine, increased the β-amyloid load in mouse brain microvessels [176]. Conversely, the overexpression of pro-inflammatory cytokines clearly reduced β-amyloid deposition in transgenic AD mice [211]. These studies indicate that the presence of immunosuppressive microenvironment in AD brain induced by AhR signaling, probably as a counteracting response to inflammatory insults (see above), disturbs the clearance of β-amyloid deposits.

## AhR signaling impairs circadian regulation and integrity of BBB

There is clear evidence that the gut microbiota regulates circadian rhythms of the host and vice versa, i.e., there exists a bidirectional circadian balance between the gut microbiota and the host tissues [212]. In fact, microbial oscillations regulate host circadian clocks, whereas host circadian cycles modulate microbial rhythms and even microbial composition. Moreover, dietary interventions are able to control host circadian rhythms via the gut microbiota [213]. Recently, Petrus et al. [214] demonstrated that tryptophan metabolites affected both the central and peripheral circadian clocks in mice although the mechanisms need to be clarified. In the circadian system, the master pacemaker in mammals is the suprachiasmatic nucleus (SCN) in the hypothalamus which is coupled with neuronal circadian oscillators [215]. The peripheral system contains diverse cell-autonomous circadian clocks which control many physiological functions, e.g., cellular metabolism under hormonal regulation. It is known that the gut microbiota coordinates diurnal rhythms of innate immunity through the control of circadian clocks [216] and it seems that circadian rhythms control the activity of the microbiota-gut-brain axis [217]. Interestingly, there is convincing evidence that the regulation of normal circadian rhythmicity is disrupted in AD patients [218]. Disturbances in the sleep–wake cycle are one of the best known of many physiological effects induced by dysfunctions of circadian rhythms. Insomnia aggravates several pathological processes linked to AD, e.g., β-amyloid accumulation. Given that waste material clearance from the brain occurs during sleep, it seems probable that the circadian system also controls the glymphatic flow from the brain [219]. The glymphatic system, formed by astrocytes, is a fluid transportation system, similar to the lymphatic system in other tissues [220]. Many toxic compounds, such as β-amyloid and tau proteins, are cleared through the glymphatic system. There is accumulating evidence that the glymphatic system is impaired in AD, especially the function of aquaporin-4 (AQP4) water channels [221, 222].

There are many studies indicating that the signaling of AhR is subjected to circadian regulation [63, 223]. For instance, the effects of AhR agonists on the expression of AhR-driven genes are dependent on diurnal variation. Interestingly, the major genes of circadian regulations, i.e., the *BMAL1*, *CLOCK*, and *PER1/2*, are members of the PAS family, similarly to the *AhR* gene [223]. Moreover, the binding partner of the AhR protein, the ARNT protein (see above), shares similar domains as the BMAL1 factor indicating that the AhR protein can heterodimerize with BMAL1 and thus affect the circadian regulation. Several studies have revealed that the AhR factor heterodimerizes with the BMAL1 protein, thus significantly attenuating the BMAL1/CLOCK-driven regulation of circadian genes, e.g., that of the *PER1* gene, a repressor of BMAL1/CLOCK transcription [224]. For instance, Fader et al. [225] demonstrated that exposure to TCDD, an agonist of the Ah receptor, abolished the circadian regulation of hepatic metabolic activity in mice. Many review articles have elucidated the impact of AhR-induced disruption of circadian rhythms to physiological and pathological processes [223]. There are observations indicating that the expression of BMAL1 is regulated through the DNA methylation sites in the promoter of the *BMAL1* gene [226]. Cronin et al. [226] reported that aberrant DNA methylation appeared during the early stages of AD, probably affecting the circadian alterations observed in AD. Interestingly, Nakazato et al. [227] demonstrated that a deletion of the *Bmal1* gene in mice caused hyperpermeability in the BBB which was associated with the loss of pericytes in the BBB. They also reported that the deletion of the *Bmal1* gene induced a robust activation of astrocytes associated with a clear decline in the expression of AQP4 in the end-feet processes of perivascular astrocytes. This indicates that a deficiency of BMAL1 factor probably affects glymphatic flow which is under the circadian regulation. Recently, Zhang et al. [228] demonstrated that the circadian clocks control the permeability and the efflux of xenobiotics in mouse BBB. Cuddapah et al. [229] have reviewed the evidence on the circadian regulation of the BBB. Currently, it needs to be clarified whether the gut microbiota-driven regulation of brain circadian rhythms is actually mediated by the AhR-induced control of BMAL1/CLOCK signaling.

## Conclusions

The etiology of AD pathology is still elusive although there are extensive research enterprises focusing on molecular genetics and therapeutic treatments. Molecular studies have revealed that the activation of immune system has a crucial role in the pathogenesis of AD [230]. In AD brain, neuroinflammation and the activation of microglia are common molecular hallmarks although the cause of inflammatory changes still needs to be clarified. Interestingly, there is accumulating evidence that chronic systemic inflammatory conditions seem to promote the inflammatory state in the AD brain [231]. There is abundant evidence that chronic kidney disease is associated with AD pathology [119]. Moreover, inflammatory bowel disease and periodontitis were shown to be associated with the increased risk for dementia [103, 232]. Recent studies have revealed that gut microbiota can control brain homeostasis and the dysbiosis of microbiota induces many brain disorders via the gut-brain signaling axis. It is known that tryptophan metabolites are important messengers involved in the communication between the gastrointestinal tract and the brain. Interestingly, many tryptophan metabolites are agonists of AhR factor, and thus, they are able to activate AhR signaling in the brain. The expression of AhR factor is enriched in the barrier organs involving also the BBB in the brain. It is known that AhR factor has a major role in many vascular diseases, such as atherosclerosis, hypertension, and hyperlipidemia. There is convincing evidence that the structural and functional properties of the BBB are impaired in the brains of AD patients. The activation of AhR signaling stimulates the renin-angiotensin system in the brain inducing a local hypoperfusion. AhR signaling also inhibits the activity of eNOS which suppresses NO production and thus prevents vascular dilatation. Both of these processes expose microvessels and brain tissues to the risk of suffering hypoxic injuries. AhR signaling also provokes several stresses, such as oxidative and nitrosative stresses and energy metabolic shortage. These stresses enhance not only the stiffness and calcification of the BBB but they also promote cellular senescence in the cells of vascular wall. It is evident that treatments with AhR agonists can increase the permeability of the BBB. Moreover, AhR signaling disturbs the maintenance of circadian rhythms because Ah receptor can heterodimerize with BMAL1 protein and thus interfere with circadian signaling. The AhR-induced disruption of circadian clocks impairs the functional properties of the BBB, probably also disturbing glymphatic flow. In summary, it seems plausible that a dysbiosis of gut microbiota can disturb the function of the BBB via the activation of AhR signaling and thus aggravate AD pathology.

## References

[1] Grice EA, Segre JA (2012). The human microbiome: our second genome. Annu Rev Genomics Hum Genet.

[2] Li C, Liang Y, Qiao Y (2022) Messengers from the gut: gut microbiota-derived metabolites on host regulation. Front Microbiol 13:863407. 10.3389/fmicb.2022.863407 10.3389/fmicb.2022.863407 PMC9073088 35531300

[3] Doifode T, Giridharan VV, Generoso JS, Bhatti G, Collodel A, Schulz PE, Forlenza OV, Barichello T (2021) The impact of the microbiota-gut-brain axis on Alzheimer’s disease pathophysiology. Pharmacol Res 164:105314. 10.1016/j.phrs.2020.105314 10.1016/j.phrs.2020.105314 33246175

[4] Leblhuber F, Ehrlich D, Steiner K, Geisler S, Fuchs D, Lanser L, Kurz K (2021). The immunopathogenesis of Alzheimer’s disease is related to the composition of gut microbiota. Nutrients.

[5] Bairamian D, Sha S, Rolhion N, Sokol H, Dorothee G, Lemere CA, Krantic S (2022). Microbiota in neuroinflammation and synaptic dysfunction: a focus on Alzheimer’s disease. Mol Neurodegener.

[6] Dong F, Perdew GH (2020). The aryl hydrocarbon receptor as a mediator of host-microbiota interplay. Gut Microbes.

[7] Ma N, He T, Johnston LJ, Ma X (2020). Host-microbiome interactions: the aryl hydrocarbon receptor as a critical node in tryptophan metabolites to brain signaling. Gut Microbes.

[8] Lee HU, McPherson ZE, Tan B, Korecka A, Pettersson S (2017). Host-microbiome interactions: the aryl hydrocarbon receptor and the central nervous system. J Mol Med (Berl).

[9] Salminen A (2022). Aryl hydrocarbon receptor (AhR) reveals evidence of antagonistic pleiotropy in the regulation of the aging process. Cell Mol Life Sci.

[10] Ramos-Garcia NA, Orozco-Ibarra M, Estudillo E, Elizondo G, Gomez Apo E, Chavez Macias LG, Sosa-Ortiz AL, Torres-Ramos MA (2020). Aryl hydrocarbon receptor in post-mortem hippocampus and in serum from young, elder, and Alzheimer’s patients. Int J Mol Sci.

[11] Xiao L, Zhang Z, Luo X (2014). Roles of xenobiotic receptors in vascular pathophysiology. Circ J.

[12] Zlokovic BV (2011). Neurovascular pathways to neurodegeneration in Alzheimer’s disease and other disorders. Nat Rev Neurosci.

[13] Huang Z, Wong LW, Su Y, Huang X, Wang N, Chen H, Yi C (2020) Blood-brain barrier integrity in the pathogenesis of Alzheimer’s disease. Front Neuroendocrinol 59:100857. 10.1016/j.yfrne.2020.100857 10.1016/j.yfrne.2020.100857 32781194

[14] Hahn ME, Karchner SI, Merson RR (2017). Diversity as opportunity: insights from 600 million years of AHR evolution. Curr Opin Toxicol.

[15] Nebert DW (2017). Aryl hydrocarbon receptor (AHR): “pioneer member” of the basic-helix/loop/helix per-Arnt-sim (bHLH/PAS) family of “sensors” of foreign and endogenous signals. Prog Lipid Res.

[16] Xue Z, Li D, Yu W, Zhang Q, Hou X, He Y, Kou X (2017). Mechanisms and therapeutic prospects of polyphenols as modulators of the aryl hydrocarbon receptor. Food Funct.

[17] McMillan BJ, Bradfield CA (2007). The aryl hydrocarbon receptor is activated by modified low-density lipoprotein. Proc Natl Acad Sci USA.

[18] Stockinger B, Di Meglio P, Gialitakis M, Duarte JH (2014). The aryl hydrocarbon receptor: multitasking in the immune system. Annu Rev Immunol.

[19] Vogel CF, Ishihara Y, Campbell CE, Kado SY, Nguyen-Chi A, Sweeney C, Pollet M, Haarmann-Stemmann T, Tuscano JM (2019). A protective role of aryl hydrocarbon receptor repressor in inflammation and tumor growth. Cancers (Basel).

[20] Vogel CF, Sciullo E, Li W, Wong P, Lazennec G, Matsumura F (2007). RelB, a new partner of aryl hydrocarbon receptor-mediated transcription. Mol Endocrinol.

[21] Marlowe JL, Fan Y, Chang X, Peng L, Knudsen ES, Xia Y, Puga A (2008). The aryl hydrocarbon receptor binds to E2F1 and inhibits E2F1-induced apoptosis. Mol Biol Cell.

[22] Tomkiewicz C, Herry L, Bui LC, Metayer C, Bourdeloux M, Barouki R, Coumoul X (2013). The aryl hydrocarbon receptor regulates focal adhesion sites through a non-genomic FAK/Src pathway. Oncogene.

[23] Pallotta MT, Fallarino F, Matino D, Macchiarulo A, Orabona C (2014). AhR-mediated, non-genomic modulation of IDO1 function. Front Immunol.

[24] Esser C, Rannug A (2015). The aryl hydrocarbon receptor in barrier organ physiology, immunology, and toxicology. Pharmacol Rev.

[25] Lamas B, Natividad JM, Sokol H (2018). Aryl hydrocarbon receptor and intestinal immunity. Mucosal Immunol.

[26] Nacarino-Palma A, Gonzalez-Rico FJ, Rejano-Gordillo CM, Ordiales-Talavero A, Merino JM, Fernandez-Salguero PM (2021). The aryl hydrocarbon receptor promotes differentiation during mouse preimplantational embryo development. Stem Cell Reports.

[27] Vaughan KL, Franchini AM, Kern HG, Lawrence BP (2021). The aryl hydrocarbon receptor modulates murine hematopoietic stem cell homeostasis and influences lineage-biased stem and progenitor cells. Stem Cells Dev.

[28] Wei GZ, Martin KA, Xing PY, Agrawal R, Whiley L, Wood TK, Hejndorf S, Ng YZ, Low JZY, Rossant J et al (2021) Tryptophan-metabolizing gut microbes regulate adult neurogenesis via the aryl hydrocarbon receptor. Proc Natl Acad Sci USA 118:e2021091118. 10.1073/pnas.2021091118 10.1073/pnas.2021091118 PMC8271728 34210797

[29] Kim HR, Kang SY, Kim HO, Park CW, Chung BY (2020). Role of aryl hydrocarbon receptor activation and autophagy in psoriasis-related inflammation. Int J Mol Sci.

[30] Kondrikov D, Elmansi A, Bragg RT, Mobley T, Barrett T, Eisa N, Kondrikova G, Schoeinlein P, Aguilar-Perez A, Shi XM, Fulzele S et al (2020) Kynurenine inhibits autophagy and promotes senescence in aged bone marrow mesenchymal stem cells through the aryl hydrocarbon receptor pathway. Exp Gerontol 130:110805. 10.1016/j.exger.2019.110805 10.1016/j.exger.2019.110805 PMC7861134 31812582

[31] Hillegass JM, Murphy KA, Villano CM, White LA (2006). The impact of aryl hydrocarbon receptor signaling on matrix metabolism: implications for development and disease. Biol Chem.

[32] Eckers A, Jakob S, Heiss C, Haarmann-Stemmann T, Goy C, Brinkmann V, Cortese-Krott MM, Sansone R, Esser C, Ale-Agha N, Altschmied J, Ventura N, Haendeler J (2016). The aryl hydrocarbon receptor promotes aging phenotypes across species. Sci Rep.

[33] Ojo ES, Tischkau SA (2021). The Role of AhR in the hallmarks of brain aging: friend and foe. Cells.

[34] Mayer EA, Tillisch K, Gupta A (2015). Gut/brain axis and the microbiota. J Clin Invest.

[35] Bonaz B, Bazin T, Pellissier S (2018). The Vagus nerve at the interface of the microbiota-gut-brain axis. Front Neurosci.

[36] Frankiensztajn LM, Elliott E, Koren O (2020). The microbiota and the hypothalamus-pituitary-adrenocortical (HPA) axis, implications for anxiety and stress disorders. Curr Opin Neurobiol.

[37] Gao J, Xu K, Liu H, Liu G, Bai M, Peng C, Li T, Yin Y (2018). Impact of the gut microbiota on intestinal immunity mediated by tryptophan metabolism. Front Cell Infect Microbiol.

[38] Jackson MA, Verdi S, Maxan ME, Shin CM, Zierer J, Bowyer RCE, Martin T, Williams FMK, Menni C, Bell JT, Spector TD, Steves CJ (2018). Gut microbiota associations with common diseases and prescription medications in a population-based cohort. Nat Commun.

[39] Park J, Kim CH (2021). Regulation of common neurological disorders by gut microbial metabolites. Exp Mol Med.

[40] Wu L, Han Y, Zheng Z, Peng G, Liu P, Yue S, Zhu S, Chen J, Lv H, Shao L, Sheng Y, Wang Y, Li L, Li L, Wang B (2021). Altered gut microbial metabolites in amnestic mild cognitive impairment and Alzheimer’s disease: signals in host-microbe interplay. Nutrients.

[41] Aoki R, Aoki-Yoshida A, Suzuki C, Takayama Y (2018). Indole-3-pyruvic acid, an aryl hydrocarbon receptor activator, suppresses experimental colitis in mice. J Immunol.

[42] van der Velpen V, Teav T, Gallart-Ayala H, Mehl F, Konz I, Clark C, Oikonomidi A, Peyratout G, Henry H, Delorenzi M, Ivanisevic J, Popp J (2019). Systemic and central nervous system metabolic alterations in Alzheimer’s disease. Alzheimers Res Ther.

[43] Whiley L, Chappell KE, D'Hondt E, Lewis MR, Jimenez B, Snowden SG, Soininen H, Kloszewska I, Mecocci P, Tsolaki M, Vellas B, Swann JR, Hye A, Lovestone S, Legido-Quigley C, Holmes E, AddNeuroMed consortium,  (2021). Metabolic phenotyping reveals a reduction in the bioavailability of serotonin and kynurenine pathway metabolites in both the urine and serum of individuals living with Alzheimer’s disease. Alzheimers Res Ther.

[44] Teruya T, Chen YJ, Kondoh H, Fukuji Y, Yanagida M (2021) Whole-blood metabolomics of dementia patients reveal classes of disease-linked metabolites. Proc Natl Acad Sci USA 118:e2022857118. 10.1073/pnas.2022857118 10.1073/pnas.2022857118 PMC8449400 34493657

[45] Zhuang ZQ, Shen LL, Li WW, Fu X, Zeng F, Gui L, Lü Y, Cai M, Zhu C, Tan YL, Zheng P, Li HY, Zhu J, Zhou HD, Bu XL, Wang YJ (2018). Gut microbiota is altered in patients with Alzheimer’s disease. J Alzheimers Dis.

[46] Harach T, Marungruang N, Duthilleul N, Cheatham V, Mc Coy KD, Frisoni G, Neher JJ, Fak F, Jucker M, Lasser T, Bolmont T (2017). Reduction of Aβ amyloid pathology in APPPS1 transgenic mice in the absence of gut microbiota. Sci Rep.

[47] Kim MS, Kim Y, Choi H, Kim W, Park S, Lee D, Kim DK, Kim HJ, Choi H, Hyun DW, Lee JY, Choi EY, Lee DS, Bae JW, Mook-Jung I (2020). Transfer of a healthy microbiota reduces amyloid and tau pathology in an Alzheimer’s disease animal model. Gut.

[48] Minter MR, Zhang C, Leone V, Ringus DL, Zhang X, Oyler-Castrillo P, Musch MW, Liao F, Ward JF, Holtzman DM, Chang EB, Tanzi RE, Sisodia SS (2016). Antibiotic-induced perturbations in gut microbial diversity influences neuro-inflammation and amyloidosis in a murine model of Alzheimer’s disease. Sci Rep.

[49] Ligezka AN, Sonmez AI, Corral-Frias MP, Golebiowski R, Lynch B, Croarkin PE, Romanowicz M (2021) A systematic review of microbiome changes and impact of probiotic supplementation in children and adolescents with neuropsychiatric disorders. Prog Neuropsychopharmacol Biol Psychiatry 108:110187. 10.1016/j.pnpbp.2020.110187 10.1016/j.pnpbp.2020.110187 PMC8138744 33271210

[50] Xiang S, Ji JL, Li S, Cao XP, Xu W, Tan L, Tan CC (2022) Efficacy and safety of probiotics for the treatment of Alzheimer’s disease, mild cognitive impairment, and Parkinson’s disease: a systematic review and meta-analysis. Front Aging Neurosci 14:730036. 10.3389/fnagi.2022.730036 10.3389/fnagi.2022.730036 PMC8851038 35185522

[51] de Rijke TJ, Doting MHE, van Hemert S, De Deyn PP, van Munster BC, Harmsen HJM, Sommer IEC (2022) A systematic review on the effects of different types of probiotics in animal Alzheimer’s disease studies. Front Psychiatry 13:879491. 10.3389/fpsyt.2022.879491 10.3389/fpsyt.2022.879491 PMC9094066 35573324

[52] Frausto DM, Forsyth CB, Keshavarzian A, Voigt RM (2021) Dietary regulation of gut-brain axis in Alzheimer’s disease: Importance of microbiota metabolites. Front Neurosci 15:736814 10.3389/fnins.2021.736814 10.3389/fnins.2021.736814 PMC8639879 34867153

[53] Kang JW, Zivkovic AM (2021). The potential utility of prebiotics to modulate Alzheimer’s disease: a review of the evidence. Microorganisms.

[54] Scarmeas N, Stern Y, Tang MX, Mayeux R, Luchsinger JA (2006). Mediterranean diet and risk for Alzheimer’s disease. Ann Neurol.

[55] Merra G, Noce A, Marrone G, Cintoni M, Tarsitano MG, Capacci A, De Lorenzo A (2020). Influence of Mediterranean diet on human gut microbiota. Nutrients.

[56] Dong F, Hao F, Murray IA, Smith PB, Koo I, Tindall AM, Kris-Etherton PM, Gowda K, Amin SG, Patterson AD, Perdew GH (2020). Intestinal microbiota-derived tryptophan metabolites are predictive of Ah receptor activity. Gut Microbes.

[57] Agus A, Planchais J, Sokol H (2018). Gut microbiota regulation of tryptophan metabolism in health and disease. Cell Host Microbe.

[58] Roth W, Zadeh K, Vekariya R, Ge Y, Mohamadzadeh M (2021). Tryptophan metabolism and gut-brain homeostasis. Int J Mol Sci.

[59] Hwang IK, Yoo KY, Li H, Park OK, Lee CH, Choi JH, Jeong YG, Lee YL, Kim YM, Kwon YG, Won MH (2009). Indole-3-propionic acid attenuates neuronal damage and oxidative stress in the ischemic hippocampus. J Neurosci Res.

[60] Hendrikx T, Schnabl B (2019). Indoles: metabolites produced by intestinal bacteria capable of controlling liver disease manifestation. J Intern Med.

[61] Lee JH, Lee J (2010). Indole as an intercellular signal in microbial communities. FEMS Microbiol Rev.

[62] Rannug A, Rannug U (2018). The tryptophan derivative 6-formylindolo[3,2-b]carbazole, FICZ, a dynamic mediator of endogenous aryl hydrocarbon receptor signaling, balances cell growth and differentiation. Crit Rev Toxicol.

[63] Rannug A (2020). How the AHR became important in intestinal homeostasis-a diurnal FICZ/AHR/CYP1A1 feedback controls both immunity and immunopathology. Int J Mol Sci.

[64] Smirnova A, Wincent E, Vikström Bergander L, Alsberg T, Bergman J, Rannug A, Rannug U (2016). Evidence for new light-independent pathways for generation of the endogenous aryl hydrocarbon receptor agonist FICZ. Chem Res Toxicol.

[65] Bergander L, Wincent E, Rannug A, Foroozesh M, Alworth W, Rannug U (2004). Metabolic fate of the Ah receptor ligand 6-formylindolo[3,2-b]carbazole. Chem Biol Interact.

[66] Wincent E, Bengtsson J, Mohammadi Bardbori A, Alsberg T, Luecke S, Rannug U, Rannug A (2012). Inhibition of cytochrome P4501-dependent clearance of the endogenous agonist FICZ as a mechanism for activation of the aryl hydrocarbon receptor. Proc Natl Acad Sci USA.

[67] Keshavarzi M, Khoshnoud MJ, Ghaffarian Bahraman A, Mohammadi-Bardbori A (2020). An endogenous ligand of aryl hydrocarbon receptor 6-formylindolo[3,2-b]carbazole (FICZ) is a signaling molecule in neurogenesis of adult hippocampal neurons. J Mol Neurosci.

[68] Tanaka Y, Uchi H, Hashimoto-Hachiya A, Furue M (2018). Tryptophan photoproduct FICZ upregulates IL1A, IL1B, and IL6 expression via oxidative stress in keratinocytes. Oxid Med Cell Longev.

[69] Murai M, Tsuji G, Hashimoto-Hachiya A, Kawakami Y, Furue M, Mitoma C (2018). An endogenous tryptophan photo-product, FICZ, is potentially involved in photo-aging by reducing TGF-β-regulated collagen homeostasis. J Dermatol Sci.

[70] Kim DJ, Iwasaki A, Chien AL, Kang S (2022) UVB-mediated DNA damage induces matrix metalloproteinases to promote photoaging in an AhR- and SP1-dependent manner. JCI Insight 7:e156344. 10.1172/jci.insight.156344 10.1172/jci.insight.156344 PMC9090247 35316219

[71] Marszalek-Grabska M, Walczak K, Gawel K, Wicha-Komsta K, Wnorowska S, Wnorowski A, Turski WA (2021) Kynurenine emerges from the shadows - current knowledge on its fate and function. Pharmacol Ther 225:107845. 10.1016/j.pharmthera.2021.107845 10.1016/j.pharmthera.2021.107845 33831481

[72] Lovelace MD, Varney B, Sundaram G, Lennon MJ, Lim CK, Jacobs K, Guillemin GJ, Brew BJ (2017). Recent evidence for an expanded role of the kynurenine pathway of tryptophan metabolism in neurological diseases. Neuropharmacology.

[73] Savitz J (2020). The kynurenine pathway: a finger in every pie. Mol Psychiatry.

[74] DiNatale BC, Murray IA, Schroeder JC, Flaveny CA, Lahoti TS, Laurenzana EM, Omiecinski CJ, Perdew GH (2010). Kynurenic acid is a potent endogenous aryl hydrocarbon receptor ligand that synergistically induces interleukin-6 in the presence of inflammatory signaling. Toxicol Sci.

[75] Mezrich JD, Fechner JH, Zhang X, Johnson BP, Burlingham WJ, Bradfield CA (2010). An interaction between kynurenine and the aryl hydrocarbon receptor can generate regulatory T cells. J Immunol.

[76] Baumgartner R, Forteza MJ, Ketelhuth DFJ (2019) The interplay between cytokines and the kynurenine pathway in inflammation and atherosclerosis. Cytokine 122:154148. 10.1016/j.cyto.2017.09.004 10.1016/j.cyto.2017.09.004 28899580

[77] Braidy N, Guillemin GJ, Mansour H, Chan-Ling T, Grant R (2011). Changes in kynurenine pathway metabolism in the brain, liver and kidney of aged female Wistar rats. FEBS J.

[78] Salminen A (2022) Role of indoleamine 2,3-dioxygenase 1 (IDO1) and kynurenine pathway in the regulation of the aging process. Ageing Res Rev 75:101573. 10.1016/j.arr.2022.101573 10.1016/j.arr.2022.101573 35085834

[79] Chatterjee P, Zetterberg H, Goozee K, Lim CK, Jacobs KR, Ashton NJ, Hye A, Pedrini S, Sohrabi HR, Shah T, Asih PR, Dave P, Shen K, Taddei K, Lovejoy DB, Guillemin GJ, Blennow K, Martins RN (2019). Plasma neurofilament light chain and amyloid-β are associated with the kynurenine pathway metabolites in preclinical Alzheimer’s disease. J Neuroinflammation.

[80] Reigstad CS, Salmonson CE, Rainey JF, Szurszewski JH, Linden DR, Sonnenburg JL, Farrugia G, Kashyap PC (2015). Gut microbes promote colonic serotonin production through an effect of short-chain fatty acids on enterochromaffin cells. FASEB J.

[81] Yano JM, Yu K, Donaldson GP, Shastri GG, Ann P, Ma L, Nagler CR, Ismagilov RF, Mazmanian SK, Hsiao EY (2015). Indigenous bacteria from the gut microbiota regulate host serotonin biosynthesis. Cell.

[82] Ma N, Zhang J, Reiter RJ, Ma X (2020). Melatonin mediates mucosal immune cells, microbial metabolism, and rhythm crosstalk: a therapeutic target to reduce intestinal inflammation. Med Res Rev.

[83] Mandic AD, Woting A, Jaenicke T, Sander A, Sabrowski W, Rolle-Kampcyk U, von Bergen M, Blaut M (2019). Clostridium ramosum regulates enterochromaffin cell development and serotonin release. Sci Rep.

[84] Huang F, Wu X (2021) Brain neurotransmitter modulation by gut microbiota in anxiety and depression. Front Cell Dev Biol 9:649103. 10.3389/fcell.2021.649103 10.3389/fcell.2021.649103 PMC7991717 33777957

[85] Wu H, Denna TH, Storkersen JN, Gerriets VA (2019). Beyond a neurotransmitter: the role of serotonin in inflammation and immunity. Pharmacol Res.

[86] Li Y, Hu N, Yang D, Oxenkrug G, Yang Q (2017). Regulating the balance between the kynurenine and serotonin pathways of tryptophan metabolism. FEBS J.

[87] Araujo AM, Carvalho F, Bastos Mde L, Guedes de Pinho P, Carvalho M (2015). The hallucinogenic world of tryptamines: an updated review. Arch Toxicol.

[88] Mousseau DD (1993). Tryptamine: a metabolite of tryptophan implicated in various neuropsychiatric disorders. Metab Brain Dis.

[89] Paley EL, Denisova G, Sokolova O, Posternak N, Wang X, Brownell AL (2007). Tryptamine induces tryptophanyl-tRNA synthetase-mediated neurodegeneration with neurofibrillary tangles in human cell and mouse models. Neuromolecular Med.

[90] Dopkins N, Becker W, Miranda K, Walla M, Nagarkatti P, Nagarkatti M (2021) Tryptamine attenuates experimental multiple sclerosis through activation of aryl hydrocarbon receptor. Front Pharmacol 11:619265. 10.3389/fphar.2020.619265 10.3389/fphar.2020.619265 PMC7868334 33569008

[91] Hubbard TD, Murray IA, Perdew GH (2015). Indole and tryptophan metabolism: endogenous and dietary routes to Ah receptor activation. Drug Metab Dispos.

[92] Cheng Y, Jin UH, Allred CD, Jayaraman A, Chapkin RS, Safe S (2015). Aryl hydrocarbon receptor activity of tryptophan metabolites in young adult mouse colonocytes. Drug Metab Dispos.

[93] Vyhlidalova B, Krasulova K, Pecinkova P, Marcalikova A, Vrzal R, Zemankova L, Vanco J, Travnicek Z, Vondracek J, Karasova M, Mani S, Dvorak Z (2020). Gut microbial catabolites of tryptophan are ligands and agonists of the aryl hydrocarbon receptor: a detailed characterization. Int J Mol Sci.

[94] Jin UH, Lee SO, Sridharan G, Lee K, Davidson LA, Jayaraman A, Chapkin RS, Alaniz R, Safe S (2014). Microbiome-derived tryptophan metabolites and their aryl hydrocarbon receptor-dependent agonist and antagonist activities. Mol Pharmacol.

[95] Vogel CF, Goth SR, Dong B, Pessah IN, Matsumura F (2008). Aryl hydrocarbon receptor signaling mediates expression of indoleamine 2,3-dioxygenase. Biochem Biophys Res Commun.

[96] Manzella CR, Ackerman M, Singhal M, Ticho AL, Ceh J, Alrefai WA, Saksena S, Dudeja PK, Gill RK (2020) Serotonin modulates AhR activation by interfering with CYP1A1-mediated clearance of AhR ligands. Cell Physiol Biochem 54:126–141. 10.33594/000000209 10.33594/000000209 PMC7050772 32017483

[97] Ciorba MA (2013). Indoleamine 2,3 dioxygenase in intestinal disease. Curr Opin Gastroenterol.

[98] Sofia MA, Ciorba MA, Meckel K, Lim CK, Guillemin GJ, Weber CR, Bissonnette M, Pekow JR (2018). Tryptophan metabolism through the kynurenine pathway is associated with endoscopic inflammation in ulcerative colitis. Inflamm Bowel Dis.

[99] Forrest CM, Gould SR, Darlington LG, Stone TW (2003). Levels of purine, kynurenine and lipid peroxidation products in patients with inflammatory bowel disease. Adv Exp Med Biol.

[100] Gupta NK, Thaker AI, Kanuri N, Riehl TE, Rowley CW, Stenson WF, Ciorba MA (2012). Serum analysis of tryptophan catabolism pathway: correlation with Crohn’s disease activity. Inflamm Bowel Dis.

[101] Yu J, Wang Y, Yan F, Zhang P, Li H, Zhao H, Yan C, Yan F, Ren X (2014). Noncanonical NF-κB activation mediates STAT3-stimulated IDO upregulation in myeloid-derived suppressor cells in breast cancer. J Immunol.

[102] Fukui S, Schwarcz R, Rapoport SI, Takada Y, Smith QR (1991). Blood-brain barrier transport of kynurenines: implications for brain synthesis and metabolism. J Neurochem.

[103] Zhang B, Wang HE, Bai YM, Tsai SJ, Su TP, Chen TJ, Wang YP, Chen MH (2021). Inflammatory bowel disease is associated with higher dementia risk: a nationwide longitudinal study. Gut.

[104] Aggarwal M, Alkhayyat M, Abou Saleh M, Sarmini MT, Singh A, Garg R, Garg P, Mansoor E, Padival R, Cohen BL (2022). Alzheimer disease occurs more frequently in patients with inflammatory bowel disease: insight from a nationwide study. J Clin Gastroenterol.

[105] Kim GH, Lee YC, Kim TJ, Kim ER, Hong SN, Chang DK, Kim YH (2022). Risk of neurodegenerative diseases in patients with inflammatory bowel disease: a nationwide population-based cohort study. J Crohns Colitis.

[106] Owe-Young R, Webster NL, Mukhtar M, Pomerantz RJ, Smythe G, Walker D, Armati PJ, Crowe SM, Brew BJ (2008). Kynurenine pathway metabolism in human blood-brain-barrier cells: implications for immune tolerance and neurotoxicity. J Neurochem.

[107] Zhang Q, Sun Y, He Z, Xu Y, Li X, Ding J, Lu M, Hu G (2020). Kynurenine regulates NLRP2 inflammasome in astrocytes and its implications in depression. Brain Behav Immun.

[108] Kennedy PJ, Cryan JF, Dinan TG, Clarke G (2017). Kynurenine pathway metabolism and the microbiota-gut-brain axis. Neuropharmacology.

[109] Banoglu E, Jha GG, King RS (2001). Hepatic microsomal metabolism of indole to indoxyl, a precursor of indoxyl sulfate. Eur J Drug Metab Pharmacokinet.

[110] Banoglu E, King RS (2002). Sulfation of indoxyl by human and rat aryl (phenol) sulfotransferases to form indoxyl sulfate. Eur J Drug Metab Pharmacokinet.

[111] Tan X, Cao X, Zou J, Shen B, Zhang X, Liu Z, Lv W, Teng J, Ding X (2017). Indoxyl sulfate, a valuable biomarker in chronic kidney disease and dialysis. Hemodial Int.

[112] Rysz J, Franczyk B, Lawinski J, Olszewski R, Cialkowska-Rysz A, Gluba-Brzozka A (2021). The impact of CKD on uremic toxins and gut microbiota. Toxins (Basel).

[113] Carpenedo R, Mannaioni G, Moroni F (1998). Oxindole, a sedative tryptophan metabolite, accumulates in blood and brain of rats with acute hepatic failure. J Neurochem.

[114] Dou L, Sallee M, Cerini C, Poitevin S, Gondouin B, Jourde-Chiche N, Fallague K, Brunet P, Calaf R, Dussol B, Mallet B, Dignat-George F, Burtey S (2015). The cardiovascular effect of the uremic solute indole-3 acetic acid. J Am Soc Nephrol.

[115] Walker JA, Richards S, Belghasem ME, Arinze N, Yoo SB, Tashjian JY, Whelan SA, Lee N, Kolachalama VB, Francis J, Ravid K, Sherr D, Chitalia VC (2020). Temporal and tissue-specific activation of aryl hydrocarbon receptor in discrete mouse models of kidney disease. Kidney Int.

[116] Thörn M, Finnström N, Lundgren S, Rane A, Lööf L (2005). Cytochromes P450 and MDR1 mRNA expression along the human gastrointestinal tract. Br J Clin Pharmacol.

[117] Teubner W, Meinl W, Florian S, Kretzschmar M, Glatt H (2007). Identification and localization of soluble sulfotransferases in the human gastrointestinal tract. Biochem J.

[118] Brydges CR, Fiehn O, Mayberg HS, Schreiber H, Dehkordi SM, Bhattacharyya S, Cha J, Choi KS, Craighead WE, Krishnan RR, Rush AJ, Dunlop BW, Kaddurah-Daouk R; Mood Disorders Precision Medicine Consortium (2021). Indoxyl sulfate, a gut microbiome-derived uremic toxin, is associated with psychic anxiety and its functional magnetic resonance imaging-based neurologic signature. Sci Rep.

[119] Zhang CY, He FF, Su H, Zhang C, Meng XF (2020). Association between chronic kidney disease and Alzheimer’s disease: an update. Metab Brain Dis.

[120] Nakagawa T, Hasegawa Y, Uekawa K, Kim-Mitsuyama S (2017). Chronic kidney disease accelerates cognitive impairment in a mouse model of Alzheimer’s disease, through angiotensin II. Exp Gerontol.

[121] Yang T, Richards EM, Pepine CJ, Raizada MK (2018). The gut microbiota and the brain-gut-kidney axis in hypertension and chronic kidney disease. Nat Rev Nephrol.

[122] Bobot M, Thomas L, Moyon A, Fernandez S, McKay N, Balasse L, Garrigue P, Brige P, Chopinet S, Poitevin S, Cerini C, Brunet P, Dignat-George F, Burtey S, Guillet B, Hache G (2020). Uremic toxic Blood-Brain Barrier disruption mediated by AhR activation leads to cognitive impairment during experimental renal dysfunction. J Am Soc Nephrol.

[123] Nigam SK, Bush KT, Martovetsky G, Ahn SY, Liu HC, Richard E, Bhatnagar V, Wu W (2015). The organic anion transporter (OAT) family: a systems biology perspective. Physiol Rev.

[124] Hosoya K, Tachikawa M (2011). Roles of organic anion/cation transporters at the blood-brain and blood-cerebrospinal fluid barriers involving uremic toxins. Clin Exp Nephrol.

[125] Koizumi M, Tatebe J, Watanabe I, Yamazaki J, Ikeda T, Morita T (2014). Aryl hydrocarbon receptor mediates indoxyl sulfate-induced cellular senescence in human umbilical vein endothelial cells. J Atheroscler Thromb.

[126] Opdebeeck B, D'Haese PC, Verhulst A (2020). Molecular and cellular mechanisms that induce arterial calcification by indoxyl sulfate and P-cresyl sulfate. Toxins (Basel).

[127] Cunha RSD, Santos AF, Barreto FC, Stinghen AEM (2020). How do uremic toxins affect the endothelium?. Toxins (Basel).

[128] Matsumoto T, Takayanagi K, Kojima M, Taguchi K, Kobayashi T (2019). Acute exposure to indoxyl sulfate impairs endothelium-dependent vasorelaxation in rat aorta. Int J Mol Sci.

[129] Tumur Z, Niwa T (2009). Indoxyl sulfate inhibits nitric oxide production and cell viability by inducing oxidative stress in vascular endothelial cells. Am J Nephrol.

[130] Ito S, Osaka M, Higuchi Y, Nishijima F, Ishii H, Yoshida M (2010). Indoxyl sulfate induces leukocyte-endothelial interactions through up-regulation of E-selectin. J Biol Chem.

[131] Karbowska M, Kaminski TW, Znorko B, Domaniewski T, Misztal T, Rusak T, Pryczynicz A, Guzinska-Ustymowicz K, Pawlak K, Pawlak D (2018). Indoxyl sulfate promotes arterial thrombosis in rat model via increased levels of complex TF/VII, PAI-1, platelet activation as well as decreased contents of SIRT1 and SIRT3. Front Physiol.

[132] Hung SC, Kuo KL, Huang HL, Lin CC, Tsai TH, Wang CH, Chen JW, Lin SJ, Huang PH, Tarng DC (2016). Indoxyl sulfate suppresses endothelial progenitor cell-mediated neovascularization. Kidney Int.

[133] Yisireyili M, Saito S, Abudureyimu S, Adelibieke Y, Ng HY, Nishijima F, Takeshita K, Murohara T, Niwa T (2014) Indoxyl sulfate-induced activation of (pro)renin receptor promotes cell proliferation and tissue factor expression in vascular smooth muscle cells. PLoS One 9:e109268. 10.1371/journal.pone.0109268 10.1371/journal.pone.0109268 PMC4208748 25343458

[134] Ito S, Osaka M, Edamatsu T, Itoh Y, Yoshida M (2016). Crucial role of the aryl hydrocarbon receptor (AhR) in indoxyl sulfate-induced vascular inflammation. J Atheroscler Thromb.

[135] Huc T, Nowinski A, Drapala A, Konopelski P, Ufnal M (2018). Indole and indoxyl sulfate, gut bacteria metabolites of tryptophan, change arterial blood pressure via peripheral and central mechanisms in rats. Pharmacol Res.

[136] Sun CY, Chang SC, Wu MS (2012) Uremic toxins induce kidney fibrosis by activating intrarenal renin-angiotensin-aldosterone system associated epithelial-to-mesenchymal transition. PLoS One 7:e34026. 10.1371/journal.pone.0034026 10.1371/journal.pone.0034026 PMC3316590 22479508

[137] Sun CY, Li JR, Wang YY, Lin SY, Ou YC, Lin CJ, Wang JD, Liao SL, Chen CJ (2021) Indoxyl sulfate caused behavioral abnormality and neurodegeneration in mice with unilateral nephrectomy. Aging (Albany NY) 13:6681–6701. 10.18632/aging.202523 10.18632/aging.202523 PMC7993681 33621199

[138] Silva YP, Bernardi A, Frozza RL (2020). The role of short-chain fatty acids from gut microbiota in gut-brain communication. Front Endocrinol (Lausanne).

[139] Hoyles L, Snelling T, Umlai UK, Nicholson JK, Carding SR, Glen RC, McArthur S (2018). Microbiome-host systems interactions: protective effects of propionate upon the blood-brain barrier. Microbiome.

[140] Mishra SP, Karunakar P, Taraphder S, Yadav H (2020). Free fatty acid receptors 2 and 3 as microbial metabolite sensors to shape host health: pharmacophysiological view. Biomedicines.

[141] Garrison PM, Denison MS (2000). Analysis of the murine AhR gene promoter. J Biochem Mol Toxicol.

[142] Modoux M, Rolhion N, Lefevre JH, Oeuvray C, Nadvorník P, Illes P, Emond P, Parc Y, Mani S, Dvorak Z, Sokol H (2022). Butyrate acts through HDAC inhibition to enhance aryl hydrocarbon receptor activation by gut microbiota-derived ligands. Gut Microbes.

[143] Korecka A, Dona A, Lahiri S, Tett AJ, Al-Asmakh M, Braniste V, D'Arienzo R, Abbaspour A, Reichardt N, Fujii-Kuriyama Y, Rafter J, Narbad A, Holmes E, Nicholson J, Arulampalam V, Pettersson S (2016). Bidirectional communication between the aryl hydrocarbon receptor (AhR) and the microbiome tunes host metabolism. NPJ Biofilms Microbiomes.

[144] Jin UH, Cheng Y, Park H, Davidson LA, Callaway ES, Chapkin RS, Jayaraman A, Asante A, Allred C, Weaver EA, Safe S (2017). Short chain fatty acids enhance aryl hydrocarbon (Ah) responsiveness in mouse colonocytes and caco-2 human colon cancer cells. Sci Rep.

[145] Huuskonen J, Suuronen T, Nuutinen T, Kyrylenko S, Salminen A (2004). Regulation of microglial inflammatory response by sodium butyrate and short-chain fatty acids. Br J Pharmacol.

[146] Jiang Y, Li K, Li X, Xu L, Yang Z (2021) Sodium butyrate ameliorates the impairment of synaptic plasticity by inhibiting the neuroinflammation in 5XFAD mice. Chem Biol Interact 341:109452. 10.1016/j.cbi.2021.109452 10.1016/j.cbi.2021.109452 33785315

[147] Fernando WMADB, Martins IJ, Morici M, Bharadwaj P, Rainey-Smith SR, Lim WLF, Martins RN (2020). Sodium butyrate reduces brain amyloid-β levels and improves cognitive memory performance in an Alzheimer’s disease transgenic mouse model at an early disease stage. J Alzheimers Dis.

[148] Colombo AV, Sadler RK, Llovera G, Singh V, Roth S, Heindl S, Sebastian Monasor L, Verhoeven A, Peters F, Parhizkar S et al (2021) Microbiota-derived short chain fatty acids modulate microglia and promote Aβ plaque deposition. Elife 10:e59826. 10.7554/eLife.59826 10.7554/eLife.59826 PMC8043748 33845942

[149] Marizzoni M, Cattaneo A, Mirabelli P, Festari C, Lopizzo N, Nicolosi V, Mombelli E, Mazzelli M, Luongo D, Naviglio D, Coppola L, Salvatore M, Frisoni GB (2020). Short-chain fatty acids and lipopolysaccharide as mediators between gut dysbiosis and amyloid pathology in Alzheimer’s disease. J Alzheimers Dis.

[150] Kadry H, Noorani B, Cucullo L (2020). A blood-brain barrier overview on structure, function, impairment, and biomarkers of integrity. Fluids Barriers CNS.

[151] Kisler K, Nelson AR, Montagne A, Zlokovic BV (2017). Cerebral blood flow regulation and neurovascular dysfunction in Alzheimer disease. Nat Rev Neurosci.

[152] Filbrandt CR, Wu Z, Zlokovic B, Opanashuk L, Gasiewicz TA (2004). Presence and functional activity of the aryl hydrocarbon receptor in isolated murine cerebral vascular endothelial cells and astrocytes. Neurotoxicology.

[153] Wang X, Hawkins BT, Miller DS (2011). Aryl hydrocarbon receptor-mediated up-regulation of ATP-driven xenobiotic efflux transporters at the blood-brain barrier. FASEB J.

[154] Liu H, Li Y, Lu S, Wu Y, Sahi J (2014). Temporal expression of transporters and receptors in a rat primary co-culture blood-brain barrier model. Xenobiotica.

[155] Ren R, Lu Q, Sherchan P, Fang Y, Lenahan C, Tang L, Huang Y, Liu R, Zhang JH, Zhang J et al (2021) Inhibition of aryl hydrocarbon receptor attenuates hyperglycemia-induced hematoma expansion in an intracerebral hemorrhage mouse model. J Am Heart Assoc 10:e022701. 10.1161/JAHA.121.022701 10.1161/JAHA.121.022701 PMC8751882 34622690

[156] Barisano G, Montagne A, Kisler K, Schneider JA, Wardlaw JM, Zlokovic BV (2022). Blood-brain barrier link to human cognitive impairment and Alzheimer’s disease. Nat Cardiovasc Res.

[157] Maciel RAP, Cunha RS, Busato V, Franco CRC, Gregorio PC, Dolenga CJR, Nakao LS, Massy ZA, Boullier A, Pecoits-Filho R, Stinghen AEM (2018). Uremia impacts VE-cadherin and ZO-1 expression in human endothelial cell-to-cell junctions. Toxins (Basel).

[158] Romanitan MO, Popescu BO, Spulber S, Bajenaru O, Popescu LM, Winblad B, Bogdanovic N (2010). Altered expression of claudin family proteins in Alzheimer’s disease and vascular dementia brains. J Cell Mol Med.

[159] Chang CC, Lee PS, Chou Y, Hwang LL, Juan SH (2012). Mediating effects of aryl-hydrocarbon receptor and RhoA in altering brain vascular integrity: the therapeutic potential of statins. Am J Pathol.

[160] Verclytte S, Lopes R, Lenfant P, Rollin A, Semah F, Leclerc X, Pasquier F, Delmaire C (2016). Cerebral hypoperfusion and hypometabolism detected by arterial spin labeling MRI and FDG-PET in early-onset Alzheimer’s disease. J Neuroimaging.

[161] Nielsen RB, Egefjord L, Angleys H, Mouridsen K, Gejl M, Möller A, Brock B, Braendgaard H, Gottrup H, Rungby J, Eskildsen SF, Östergaard L (2017). Capillary dysfunction is associated with symptom severity and neurodegeneration in Alzheimer’s disease. Alzheimers Dement.

[162] Kopf PG, Huwe JK, Walker MK (2008). Hypertension, cardiac hypertrophy, and impaired vascular relaxation induced by 2,3,7,8-tetrachlorodibenzo-p-dioxin are associated with increased superoxide. Cardiovasc Toxicol.

[163] Coelho NR, Matos C, Pimpao AB, Correia MJ, Sequeira CO, Morello J, Pereira SA, Monteiro EC (2021) AHR canonical pathway: in vivo findings to support novel antihypertensive strategies. Pharmacol Res 165:105407. 10.1016/j.phrs.2020.105407 10.1016/j.phrs.2020.105407 33418029

[164] Kwon YN, Kim YJ (2021). Gut-brain-microbiota axis and hypertension: a literature review. Curr Pharm Des.

[165] Te Riet L, van Esch JH, Roks AJ, van den Meiracker AH, Danser AH (2015). Hypertension: renin-angiotensin-aldosterone system alterations. Circ Res.

[166] Jaworska K, Koper M, Ufnal M (2021). Gut microbiota and renin-angiotensin system: a complex interplay at local and systemic levels. Am J Physiol Gastrointest Liver Physiol.

[167] Nakagawa P, Gomez J, Grobe JL, Sigmund CD (2020). The renin-angiotensin system in the central nervous system and its role in blood pressure regulation. Curr Hypertens Rep.

[168] Shimizu H, Saito S, Higashiyama Y, Nishijima F, Niwa T (2013). CREB, NF-κB, and NADPH oxidase coordinately upregulate indoxyl sulfate-induced angiotensinogen expression in proximal tubular cells. Am J Physiol Cell Physiol.

[169] Qadri F, Culman J, Veltmar A, Maas K, Rascher W, Unger T (1993). Angiotensin II-induced vasopressin release is mediated through α1 adrenoceptors and angiotensin II AT1 receptors in the supraoptic nucleus. J Pharmacol Exp Ther.

[170] Vital SA, Terao S, Nagai M, Granger DN (2010). Mechanisms underlying the cerebral microvascular responses to angiotensin II-induced hypertension. Microcirculation.

[171] Wu H, Sun Q, Yuan S, Wang J, Li F, Gao H, Chen X, Yang R, Xu J (2022). AT1 receptors: their actions from hypertension to cognitive impairment. Cardiovasc Toxicol.

[172] Zhang M, Mao Y, Ramirez SH, Tuma RF, Chabrashvili T (2010). Angiotensin II induced cerebral microvascular inflammation and increased blood-brain barrier permeability via oxidative stress. Neuroscience.

[173] Agbor LN, Elased KM, Walker MK (2011). Endothelial cell-specific aryl hydrocarbon receptor knockout mice exhibit hypotension mediated, in part, by an attenuated angiotensin II responsiveness. Biochem Pharmacol.

[174] Romero Caimi G, Gorzalczany S, Bonazzola P, Deza Z, Roson MI, Alvarez L, Castilla R (2021). Angiotensin II type 1 receptor is involved in hypertension and vascular alterations caused by environmental toxicant hexachlorobenzene. Toxicol Rep.

[175] Ongali B, Nicolakakis N, Tong XK, Lecrux C, Imboden H, Hamel E (2018). Transforming growth factor-β1 induces cerebrovascular dysfunction and astrogliosis through angiotensin II type 1 receptor-mediated signaling pathways. Can J Physiol Pharmacol.

[176] Wyss-Coray T, Lin C, Sanan DA, Mucke L, Masliah E (2000). Chronic overproduction of transforming growth factor-β1 by astrocytes promotes Alzheimer’s disease-like microvascular degeneration in transgenic mice. Am J Pathol.

[177] Ismael S, Mirzahosseini G, Ahmed HA, Yoo A, Kassan M, Malik KU, Ishrat T (2021). Renin-angiotensin system alterations in the human Alzheimer’s disease brain. J Alzheimers Dis.

[178] Labandeira-Garcia JL, Rodríguez-Perez AI, Garrido-Gil P, Rodriguez-Pallares J, Lanciego JL, Guerra MJ (2017). Brain renin-angiotensin system and microglial polarization: implications for aging and neurodegeneration. Front Aging Neurosci.

[179] Zhu J, Song W, Li L, Fan X (2016). Endothelial nitric oxide synthase: a potential therapeutic target for cerebrovascular diseases. Mol Brain.

[180] Chang CC, Hsu YH, Chou HC, Lee YG, Juan SH (2017). 3-Methylcholanthrene/aryl-hydrocarbon receptor-mediated hypertension through eNOS inactivation. J Cell Physiol.

[181] Radi R (2018). Oxygen radicals, nitric oxide, and peroxynitrite: Redox pathways in molecular medicine. Proc Natl Acad Sci USA.

[182] Wada T, Sunaga H, Ohkawara R, Shimba S (2013). Aryl hydrocarbon receptor modulates NADPH oxidase activity via direct transcriptional regulation of p40phox expression. Mol Pharmacol.

[183] Nakagawa K, Itoya M, Takemoto N, Matsuura Y, Tawa M, Matsumura Y, Ohkita M (2021) Indoxyl sulfate induces ROS production via the aryl hydrocarbon receptor-NADPH oxidase pathway and inactivates NO in vascular tissues. Life Sci 265:118807. 10.1016/j.lfs.2020.118807 10.1016/j.lfs.2020.118807 33232689

[184] Picon-Pages P, Garcia-Buendia J, Munoz FJ (2019). Functions and dysfunctions of nitric oxide in brain. Biochim Biophys Acta Mol Basis Dis.

[185] Wilkinson IB, Franklin SS, Cockcroft JR (2004). Nitric oxide and the regulation of large artery stiffness: from physiology to pharmacology. Hypertension.

[186] Oe Y, Mitsui S, Sato E, Shibata N, Kisu K, Sekimoto A, Miyazaki M, Sato H, Ito S, Takahashi N (2021). Lack of endothelial nitric oxide synthase accelerates ectopic calcification in uremic mice fed an adenine and high phosphorus diet. Am J Pathol.

[187] Toda N, Okamura T (2012). Cerebral blood flow regulation by nitric oxide in Alzheimer’s disease. J Alzheimers Dis.

[188] Liu R, Liu H, Ha Y, Tilton RG, Zhang W (2014) Oxidative stress induces endothelial cell senescence via downregulation of Sirt6. Biomed Res Int 2014:902842. 10.1155/2014/902842 10.1155/2014/902842 PMC4138737 25162034

[189] Guzik TJ, Touyz RM (2017). Oxidative stress, inflammation, and vascular aging in hypertension. Hypertension.

[190] Dalton TP, Puga A, Shertzer HG (2002). Induction of cellular oxidative stress by aryl hydrocarbon receptor activation. Chem Biol Interact.

[191] Davalli P, Mitic T, Caporali A, Lauriola A, D'Arca D (2016). ROS, cell senescence, and novel molecular mechanisms in aging and age-related diseases. Oxid Med Cell Longev.

[192] Masai N, Tatebe J, Yoshino G, Morita T (2010). Indoxyl sulfate stimulates monocyte chemoattractant protein-1 expression in human umbilical vein endothelial cells by inducing oxidative stress through activation of the NADPH oxidase-nuclear factor-κB pathway. Circ J.

[193] Rensink AA, de Waal RM, Kremer B, Verbeek MM (2003). Pathogenesis of cerebral amyloid angiopathy. Brain Res Brain Res Rev.

[194] Sun R, He T, Pan Y, Katusic ZS (2018) Effects of senescence and angiotensin II on expression and processing of amyloid precursor protein in human cerebral microvascular endothelial cells. Aging (Albany NY) 10:100–114. 10.18632/aging.101362 10.18632/aging.101362 PMC5811245 29348391

[195] Ionescu-Tucker A, Cotman CW (2021). Emerging roles of oxidative stress in brain aging and Alzheimer’s disease. Neurobiol Aging.

[196] Ma Q (2002). Induction and superinduction of 2,3,7,8-tetrachlorodibenzo-rho-dioxin-inducible poly(ADP-ribose) polymerase: role of the aryl hydrocarbon receptor/aryl hydrocarbon receptor nuclear translocator transcription activation domains and a labile transcription repressor. Arch Biochem Biophys.

[197] Wang X, He HJ, Xiong X, Zhou S, Wang WW, Feng L, Han R, Xie CL (2021) NAD+ in Alzheimer’s disease: Molecular mechanisms and systematic therapeutic evidence obtained in vivo. Front Cell Dev Biol 9:668491. 10.3389/fcell.2021.668491 10.3389/fcell.2021.668491 PMC8369418 34414179

[198] Czubowicz K, Jesko H, Wencel P, Lukiw WJ, Strosznajder RP (2019). The role of ceramide and sphingosine-1-phosphate in Alzheimer’s disease and other neurodegenerative disorders. Mol Neurobiol.

[199] Majumder S, Kono M, Lee YT, Byrnes C, Li C, Tuymetova G, Proia RL (2020). A genome-wide CRISPR/Cas9 screen reveals that the aryl hydrocarbon receptor stimulates sphingolipid levels. J Biol Chem.

[200] Wang HC, Wong TH, Wang LT, Su HH, Yu HY, Wu AH, Lin YC, Chen HL, Suen JL, Hsu SH, Chen LC, Zhou Y, Huang SK (2019). Aryl hydrocarbon receptor signaling promotes ORMDL3-dependent generation of sphingosine-1-phosphate by inhibiting sphingosine-1-phosphate lyase. Cell Mol Immunol.

[201] Nixon RA (2007). Autophagy, amyloidogenesis and Alzheimer disease. J Cell Sci.

[202] Salminen A, Kaarniranta K, Kauppinen A, Ojala J, Haapasalo A, Soininen H, Hiltunen M (2013). Impaired autophagy and APP processing in Alzheimer’s disease: the potential role of Beclin 1 interactome. Prog Neurobiol.

[203] Yang Z, Lin P, Chen B, Zhang X, Xiao W, Wu S, Huang C, Feng D, Zhang W, Zhang J (2021). Autophagy alleviates hypoxia-induced blood-brain barrier injury via regulation of CLDN5 (claudin 5). Autophagy.

[204] Wan C, Liu J, Nie X, Zhao J, Zhou S, Duan Z, Tang C, Liang L, Xu G (2014) 2, 3, 7, 8-Tetrachlorodibenzo-P-dioxin (TCDD) induces premature senescence in human and rodent neuronal cells via ROS-dependent mechanisms. PLoS One 9:e89811. 10.1371/journal.pone.0089811 10.1371/journal.pone.0089811 PMC3933666 24587053

[205] Han X, Zhang T, Liu H, Mi Y, Gou X (2020). Astrocyte senescence and Alzheimer’s disease: a review. Front Aging Neurosci.

[206] Vogel CF, Khan EM, Leung PS, Gershwin ME, Chang WL, Wu D, Haarmann-Stemmann T, Hoffmann A, Denison MS (2014). Cross-talk between aryl hydrocarbon receptor and the inflammatory response: a role for nuclear factor-κB. J Biol Chem.

[207] Yang X, Liu H, Ye T, Duan C, Lv P, Wu X, Liu J, Jiang K, Lu H, Yang H, Xia D, Peng E, Chen Z, Tang K, Ye Z (2020). AhR activation attenuates calcium oxalate nephrocalcinosis by diminishing M1 macrophage polarization and promoting M2 macrophage polarization. Theranostics.

[208] Quintana FJ, Basso AS, Iglesias AH, Korn T, Farez MF, Bettelli E, Caccamo M, Oukka M, Weiner HL (2008). Control of T(reg) and T(H)17 cell differentiation by the aryl hydrocarbon receptor. Nature.

[209] Monsonego A, Maron R, Zota V, Selkoe DJ, Weiner HL (2001). Immune hyporesponsiveness to amyloid β-peptide in amyloid precursor protein transgenic mice: implications for the pathogenesis and treatment of Alzheimer’s disease. Proc Natl Acad Sci USA.

[210] Krabbe G, Halle A, Matyash V, Rinnenthal JL, Eom GD, Bernhardt U, Miller KR, Prokop S, Kettenmann H, Heppner FL (2013) Functional impairment of microglia coincides with β-amyloid deposition in mice with Alzheimer-like pathology. PLoS One 8:e60921. 10.1371/journal.pone.0060921 10.1371/journal.pone.0060921 PMC3620049 23577177

[211] Chakrabarty P, Herring A, Ceballos-Diaz C, Das P, Golde TE (2011). Hippocampal expression of murine TNFα results in attenuation of amyloid deposition in vivo. Mol Neurodegener.

[212] Pearson JA, Wong FS, Wen L (2020). Crosstalk between circadian rhythms and the microbiota. Immunology.

[213] Choi H, Rao MC, Chang EB (2021). Gut microbiota as a transducer of dietary cues to regulate host circadian rhythms and metabolism. Nat Rev Gastroenterol Hepatol.

[214] Petrus P, Cervantes M, Samad M, Sato T, Chao A, Sato S, Koronowski KB, Park G, Alam Y, Mejhert N et al (2022) Tryptophan metabolism is a physiological integrator regulating circadian rhythms. Mol Metab 64:101556. 10.1016/j.molmet.2022.101556 10.1016/j.molmet.2022.101556 PMC9382333 35914650

[215] Mohawk JA, Green CB, Takahashi JS (2012). Central and peripheral circadian clocks in mammals. Annu Rev Neurosci.

[216] Brooks JF, Behrendt CL, Ruhn KA, Lee S, Raj P, Takahashi JS, Hooper LV (2021). The microbiota coordinates diurnal rhythms in innate immunity with the circadian clock. Cell.

[217] Cheng WY, Ho YS, Chang RC (2022) Linking circadian rhythms to microbiome-gut-brain axis in aging-associated neurodegenerative diseases. Ageing Res Rev 78:101620. 10.1016/j.arr.2022.101620 10.1016/j.arr.2022.101620 35405323

[218] Li Y, Shao L, Mou Y, Zhang Y, Ping Y (2021). Sleep, circadian rhythm and gut microbiota: alterations in Alzheimer’s disease and their potential links in the pathogenesis. Gut Microbes.

[219] Hablitz LM, Pla V, Giannetto M, Vinitsky HS, Stæger FF, Metcalfe T, Nguyen R, Benrais A, Nedergaard M (2020). Circadian control of brain glymphatic and lymphatic fluid flow. Nat Commun.

[220] Hablitz LM, Nedergaard M (2021). The glymphatic system: a novel component of fundamental neurobiology. J Neurosci.

[221] Harrison IF, Ismail O, Machhada A, Colgan N, Ohene Y, Nahavandi P, Ahmed Z, Fisher A, Meftah S, Murray TK, Ottersen OP, Nagelhus EA, O'Neill MJ, Wells JA, Lythgoe MF (2020). Impaired glymphatic function and clearance of tau in an Alzheimer’s disease model. Brain.

[222] Silva I, Silva J, Ferreira R, Trigo D (2021). Glymphatic system, AQP4, and their implications in Alzheimer’s disease. Neurol Res Pract.

[223] Tischkau SA (2020). Mechanisms of circadian clock interactions with aryl hydrocarbon receptor signalling. Eur J Neurosci.

[224] Xu CX, Krager SL, Liao DF, Tischkau SA (2010). Disruption of CLOCK-BMAL1 transcriptional activity is responsible for aryl hydrocarbon receptor-mediated regulation of Period1 gene. Toxicol Sci.

[225] Fader KA, Nault R, Doskey CM, Fling RR, Zacharewski TR (2019). 2,3,7,8-Tetrachlorodibenzo-p-dioxin abolishes circadian regulation of hepatic metabolic activity in mice. Sci Rep.

[226] Cronin P, McCarthy MJ, Lim ASP, Salmon DP, Galasko D, Masliah E, De Jager PL, Bennett DA, Desplats P (2017). Circadian alterations during early stages of Alzheimer’s disease are associated with aberrant cycles of DNA methylation in BMAL1. Alzheimers Dement.

[227] Nakazato R, Kawabe K, Yamada D, Ikeno S, Mieda M, Shimba S, Hinoi E, Yoneda Y, Takarada T (2017). Disruption of Bmal1 impairs blood-brain barrier integrity via pericyte dysfunction. J Neurosci.

[228] Zhang SL, Lahens NF, Yue Z, Arnold DM, Pakstis PP, Schwarz JE, Sehgal A (2021). A circadian clock regulates efflux by the blood-brain barrier in mice and human cells. Nat Commun.

[229] Cuddapah VA, Zhang SL, Sehgal A (2019). Regulation of the blood-brain barrier by circadian rhythms and sleep. Trends Neurosci.

[230] Wu KM, Zhang YR, Huang YY, Dong Q, Tan L, Yu JT (2021) The role of the immune system in Alzheimer’s disease. Ageing Res Rev 70:101409. 10.1016/j.arr.2021.101409 10.1016/j.arr.2021.101409 34273589

[231] Leblhuber F, Steiner K, Geisler S, Fuchs D, Gostner JM (2020). On the possible relevance of bottom-up pathways in the pathogenesis of Alzheimer’s disease. Curr Top Med Chem.

[232] Leblhuber F, Huemer J, Steiner K, Gostner JM, Fuchs D (2020). Knock-on effect of periodontitis to the pathogenesis of Alzheimer’s disease?. Wien Klin Wochenschr.

